# Induction of Olfaction and Cancer-Related Genes in Mice Fed a High-Fat Diet as Assessed through the Mode-of-Action by Network Identification Analysis

**DOI:** 10.1371/journal.pone.0056610

**Published:** 2013-03-26

**Authors:** Youngshim Choi, Cheol-Goo Hur, Taesun Park

**Affiliations:** 1 Department of Food and Nutrition, College of Human Ecology, Yonsei University, Seoul, Korea; 2 Plant Systems Engineering Research Center, Korea Research Institute of Bioscience and Biotechnology, Daejeon, Korea; University of Maryland School of Medicine, United States of America

## Abstract

The pathophysiological mechanisms underlying the development of obesity and metabolic diseases are not well understood. To gain more insight into the genetic mediators associated with the onset and progression of diet-induced obesity and metabolic diseases, we studied the molecular changes in response to a high-fat diet (HFD) by using a mode-of-action by network identification (MNI) analysis. Oligo DNA microarray analysis was performed on visceral and subcutaneous adipose tissues and muscles of male C57BL/6N mice fed a normal diet or HFD for 2, 4, 8, and 12 weeks. Each of these data was queried against the MNI algorithm, and the lists of top 5 highly ranked genes and gene ontology (GO)-annotated pathways that were significantly overrepresented among the 100 highest ranked genes at each time point in the 3 different tissues of mice fed the HFD were considered in the present study. The 40 highest ranked genes identified by MNI analysis at each time point in the different tissues of mice with diet-induced obesity were subjected to clustering based on their temporal patterns. On the basis of the above-mentioned results, we investigated the sequential induction of distinct olfactory receptors and the stimulation of cancer-related genes during the development of obesity in both adipose tissues and muscles. The top 5 genes recognized using the MNI analysis at each time point and gene cluster identified based on their temporal patterns in the peripheral tissues of mice provided novel and often surprising insights into the potential genetic mediators for obesity progression.

## Introduction

Microarray analysis has enabled the use of whole-genome expression profiling to understand the mechanisms underlying obesity and metabolic complications and to identify key genetic mediators. Statistical approaches used to analyze microarray data can be classified into 2 major categories: methods that identify differentially expressed genes [1], [2] and those that classify genes according to the functional dependency (e.g., hierarchical clustering) [3]. Although microarray analysis has yielded some promising results, it is not a very practical method considering the fact that identification of genes directly affected by a condition is difficult from the hundreds to thousands of genes that exhibit changes in expression. To overcome this problem, Berneardo et al. developed a model-based approach that accurately distinguishes a compound's targets from the indirect responders [4]. This approach, namely, the mode-of-action by network identification (MNI), involves the reverse engineering of a network model of regulatory interactions in an organism of interest by using a training dataset of whole-genome expression profiles. The MNI algorithm has been applied successfully to identify disease mediators as well as drug targets by studying gene-expression data from yeast [4], humans (A. Ergun and J.J. Collins, unpublished data), bacteria, and other organisms (X.H., unpublished data).

Differential expression can be studied from a static or temporal viewpoint. In a static experiment, the arrays are obtained irrespective of time, essentially taking a snapshot of gene expression. On the other hand, in a temporal experiment, the arrays are collected over a time course, facilitating the study of the dynamic behavior of gene expression. Most previously obtained microarray datasets were static, that is, the results obtained on the basis of the measurement of gene expression at a single time point [5]. Since the regulation of gene expression is a dynamic process, it is important to identify and characterize the changes in gene expression over time. Therefore, numerous time-series microarray experiments have been performed to study such biological processes such as abiotic stress, disease progression, and drug responses [6]–[8].

Microarray analysis for studying the mechanisms underlying obesity was first reported by Soukas *et al*. in 2000 [9]. They used approximately 6,500 murine genes in pairs of adipose tissues in *ob/ob* mice and wild-type lean mice. Subsequently, many such studies were conducted: more than 30 microarray approaches have been exploited in assessing the changes in gene expression in the adipose tissues, liver, hypothalamus, skeletal muscles, small intestines, and kidneys of lean and obese animals or human subjects. A frequent limitation of these studies is that they are not time-resolved and do not necessarily provide information of an end-point or disease stage. Considerably less is known about the key genetic mediators of HFD-induced obesity and the dynamics of changes in metabolic processes related to this condition. To gain more insight into the genetic mediators associated with the onset and progression of diet-induced obesity and metabolic diseases, we studied the molecular changes in response to the HFD by using an integrative time-resolved approach.

## Materials and Methods

### Ethics statement

All animal experiments were performed in accordance with the Korean Food and Drug Administration (KFDA) guidelines. Protocols were reviewed and approved by the Institutional Animal Care and Use Committee (IACUC) of the Yonsei Laboratory Animal Research Center (YLARC) (Permit #: 2011-0061). All mice were maintained in the specific pathogen-free facility of the YLARC.

### Animals and diets

Five-week-old male C57BL/6N mice were obtained from Orient Bio (Gyeonggi-do, South Korea). All animals were housed in specific pathogen-free conditions, with 21±2.0°C temperature, 50±5% relative humidity, and a 12 h-light/12 h-dark cycle. From a week before the diet intervention was started, all animals were fed standard chow. At the beginning of the study, mice were divided into 2 groups: (1) control group fed the normal diet (ND, n = 40) and (2) a group fed the high-fat diet (HFD, n = 40). Mice were provided food and water *ad libitum*. The body weight and food intake were monitored throughout the study. At 2, 4, 8, and 12 weeks after the initiation of the study, 10 animals from each group were killed. Tissues were snap-frozen immediately in liquid nitrogen and stored at −80°C until further processing.

### RNA extraction for microarray analysis

Total RNA was extracted from the epididymal and subcutaneous fat tissues and gastrocnemius muscle of each mouse by using Trizol (Invitrogen, CA, USA), according to the manufacturer's recommendations. Concentrations and purity of RNA samples were determined using a Nano Drop ND-1000 spectrophotometer (Nano Drop Technologies, Inc., Wilmington, DE, USA). RNA preparations were considered suitable for array hybridization only if samples showed intact 18S and 28S rRNA bands and displayed no chromosomal peaks or RNA degradation products. The integrity of the RNA samples was determined using a Bioanalyzer 2100 System (Agilent Technologies, Palo Alto, CA, USA).

### Real-time quantitative PCR

Real-time PCR amplification was performed with the SYBR Premix Ex Taq kit (Takara, Kyoto, Japan) on a Light Cycler 2 (Roche Applied Science, Indianapolis, USA). The initial denaturation step was at 95°C for 10 s, followed by 40 cycles of amplification at 95°C for 3 s and 60°C for 40 s. mRNA expression was determined using the relative standard curve method and normalized to the housekeeping gene. The primers (sense and antisense, respectively) were as follows: Gli2, 5′- GCC AAC CAG AAC AAG CAG AA-3′, 5′- CGC TTA TGA ATG GTG ATG GG -3′; Gucy2c, 5′- GTG CGG TTA CTG CTC TTC CA -3′, 5′- TTG TCC ATC ATC AGG ACG CT -3′; Olfr1181, 5′- CCT GAC AGT CAT GGC CTT TG -3′, 5′- ACC CAG GAA GCC CAG ATA AA -3′; Atp8b3, 5′- GTT TGA GCA GGA TGT GAC CG -3′, 5′- GGC TTG CAT GAA AAT GCT GT -3′; Tmem46, 5′- TTT TCC AGC AGC AGG AGC TA -3′, 5′- GCT GAG GAG AAA AGG GAT GC -3′; Pthr2, 5′- ATG CAA GGG AGA AAC CCA TC -3′, 5′- TAG ATC CTC CCA CAC AGC CA -3′; Cdh7, 5′- TGG ACT GGG CAT TTT CAA GA -3′, 5′- GGG GAT CAG CAT CTC GAT TT -3′; Mep1b, 5′- GAT GGC CAC ATA CCA TTC CA -3′, 5′- TAA GGC GAT AGC GCT CAA AA -3′; Lamc3, 5′- GAC ATG GGC TCT TGC TAC GA -3′, 5′- CGT TCT CGA ACT CAG GCA GA -3′; GAPDH, 5′- GGA GAT TGT TGC CAT CAA CG -3′, 5′- TTT GCC GTG AGT GGA GTC AT -3′.

### Microarray hybridization and data analysis

Equal amounts of total RNA were pooled from 10 mice in each experimental group and subjected to microarray experiments in triplicate. For analysis, 2 μg total RNA was labeled and amplified using the Universal Linkage System antisense RNA (aRNA) labeling kit (Kreatech Diagnostics, Amsterdam, The Netherlands). The Cy5-labeled aRNAs were resuspended in 10 μL of hybridization solution (GenoCheck, Korea). The labeled aRNAs were hybridized to the NimbleGen mouse whole genome 12-plex array (Roche NimbleGen, Inc., WI, USA) that contained 60-mer probes representing 42,576 genes (average 3 probes per target). The arrays were scanned using a GenePix 4000B microarray scanner. The data were extracted from the scanned images using NimbleScan software version 2.4 (Roche NimbleGen), and the Robust Multichip Average algorithm was used to generate gene expression values. The normalized and log-transformed intensity values were then analyzed using GeneSpring GX 10 (Agilent Technologies, Santa Clara, CA, USA) and GenePlex (Istech, Inc., Seoul, South Korea). The details of labeling, hybridization, scanning, and normalization of the data are provided on the NimbleGen website (http://www.nimblegen.com). Gene expression levels between the ND and HFD samples were assessed by comparing the average expression ratios of each group. Hierarchical clustering was performed in GeneSpring GX 7.3.1 software (Agilent Technologies, Santa Clara, CA), using average gene expression values under HFD condition divided by the median of ND gene expression, per time-point.

### MNI algorithm

We constructed a compendium dataset consisting of hundreds of expression profiles in the organism of interest; that the expression profiles were downloaded from the Gene Expression Omnibus, a public repository of microarray studies. The MNI algorithm was applied, using the method developed by Xing et al. [10], and was configured to output the top 200 mediators for each sample and generate the associated Z-scores for those probe sets. The Z-score for probe sets that were not within the list of the top 100 probe sets identified as mediators for a given sample were set to zero. To identify a characteristic list of genes within each group, the Z-scores across samples and probe sets for corresponding genes were averaged and ranked. The top 100 genes within that list were selected to be reported as significant genetic mediators. A higher average Z-score is an indication of higher number of occurrences of a gene on the lists generated by the MNI algorithm in each group. The 100 highest ranked genes were classified according to the biological process in which they are involved as per the criteria established by the GO.

## Results

### Effect of HFD feeding on visceral adiposity

**Figure 1:**
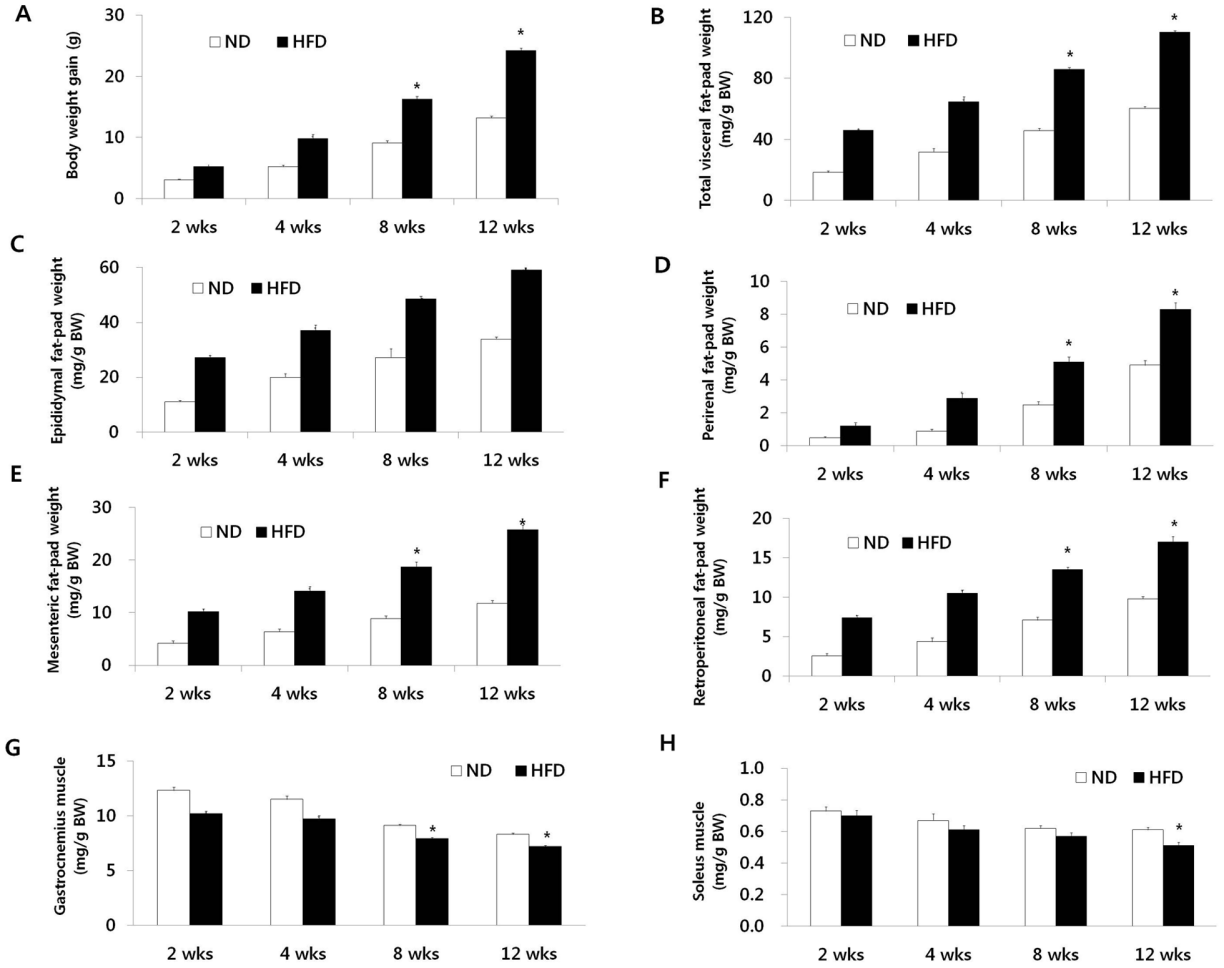
Changes in body weight, visceral fat-pad weights, and muscle masses over time. (A) Body weight gain. (B) Total visceral fat-pad weight. (C) Epididymal fat-pad weight. (D) Perirenal fat-pad weight. (E) Mesenteric fat-pad weight. (F) Retroperitoneal fat-pad weight. (G) Gastrocnemius muscle mass. (H) Soleus muscle mass. Data are presented as means ± SEM. *P<0.05.

The body weight gains of mice fed the 2 diets over the 12-week period are shown in Figure 1A. The difference in body weight between the 2 groups continued to increase over the course of experimental feeding: the difference was about 45% by 12 weeks. The increase in body weight associated with the HFD was partially attributed to the expansion of visceral adipose tissues. The masses of the epididymal, perirenal, mesenteric, and retroperitoneal fat pads of the mice fed HFD for 12 weeks were 42%, 40%, 54%, and 42%, respectively; the difference in the masses was larger in the HFD-fed mice than in the ND-fed group (Figure 1B–F). Moreover, HFD-fed mice exhibited significant reductions in the wet weights of the gastrocnemius (−13%) and soleus (−16%) muscles at 12 weeks compared with those in the ND-fed mice (Figure 1G and H).

### Transcription response of WAT and muscle to HFD during the 12-week time-course

**Figure 2:**
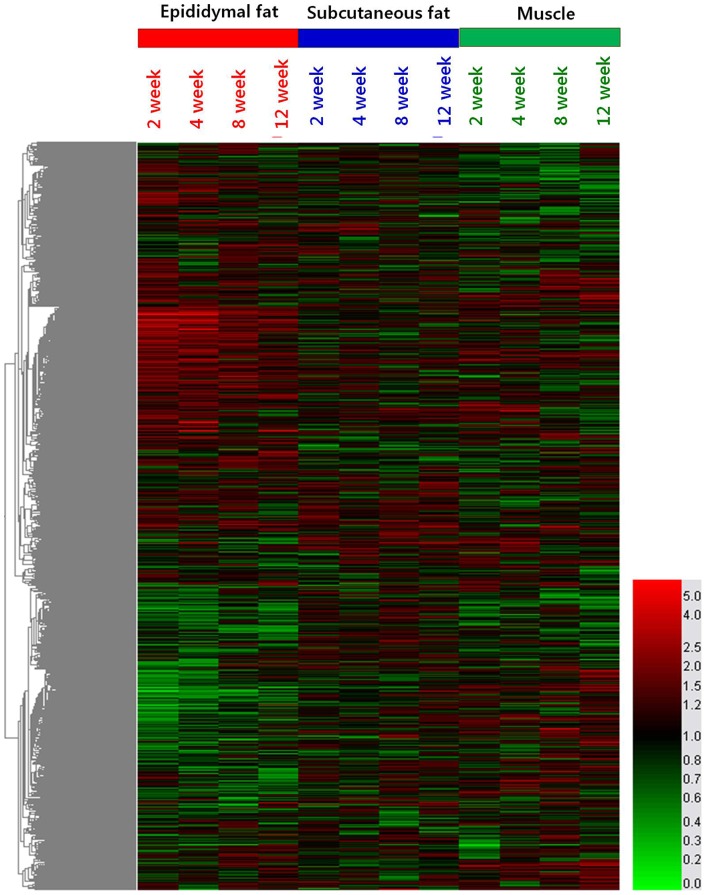
Heatmap of differentially expressed transcript sets. Values used for clustering are average HFD vs. ND per time-point expression ratio. The branches of the condition tree are colored so to discriminate three subclusters with the largest distance, corresponding to three tissues of the time-course: epididymal adipose tissue (red), subcutaneous adipose tissue (blue) and gastrocnemious muscle (green). This is summarized in the color bar underneath the cluster diagram.

Gene expression profiling in the WAT and muscle of mice was assessed through the oligonucleotide microarray analysis. Among 25,291 genes on the NimbleGen Mouse Whole Oligo 12-plex chip used in this study, 21,890 genes (86%) were identified as known genes. After determination of the temporal effects of the HFD across 12-week time-course, we focused on dissecting the HFD specific effects on the transcriptome of epididymal and subcutaneous fats and muscle. Microarray data were analyzed by hierarchical clustering of enriched functional groups of genes (based on Gene Ontology) and the major results are graphically illustrated in a heat map (Figure 2). The HFD elicited distinct changes in gene expression in epididymal and subcutaneous fats and muscle of mice over time, and most significant changes were shown in epididymal fat tissue. Specifically, prominent expression changes were observed at the early phase (week 2 to week 4) and the enrichment of lipid metabolism and inflammatory processes were significant among the up-regulated HFD-responsive genes, whereas G-protein coupled receptor protein signaling pathway and electron transport were most significant among the down-regulated HFD-responsive genes in the epididymal fat tissue.

### MNI analysis of the time course treatment with the HFD

**Figure 3:**
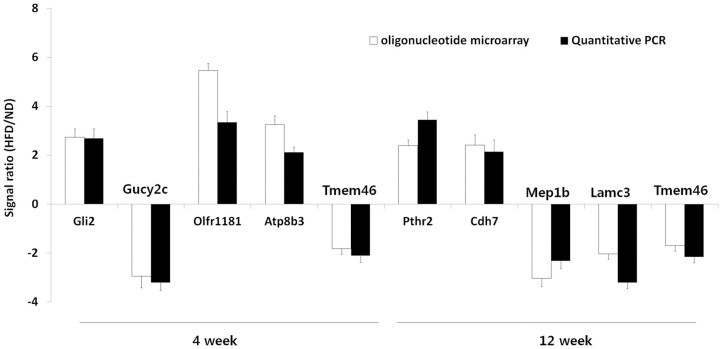
Quantitative PCR. Quantitative real-time PCR analysis of the mRNA expression on selected gene targets identified by MNI analysis in the epididymal fat tissues of mice. Results are presented as the average ± SEM of at least 3 separate experiments.

To elucidate the time course and metabolic processes underlying obesity progression induced by the HFD, we determined the gene expression profiles of the epididymal and subcutaneous fat tissues and gastrocnemius muscle of mice by using oligonucleotide microarray analysis. Each of these data was queried against the reconstructed network (MNI algorithm), and the resulting potential genetic mediators in each case were ranked according to the Z-score statistic. The lists of top 5 potential genetic mediators for obesity progression in the epididymal and subcutaneous fat tissues and gastrocnemius muscle of mice fed the HFD for 2, 4, 8, and 12 weeks are shown in Tables 1, 2, 3. The most characteristic genes across all tissues in the list were associated with cancer; the genes in this category included *Nek11*, *Gli2*, *Tmem46*, *Mep1b*, *Ccdc109b*, *Rab23*, *Patz1*, and *Hdac9*. The second representative functional theme was related to olfactory transduction, and these genes included *Olfr1181*, *Olfr1173*, *Olfr855*, *Olfr1056*, *Olfr716*, and *Tmem16b*. To validate the microarray results quantitatively, we analyzed the mRNA expression levels of top-ranked genes by real-time PCR. In all cases, a strong correspondence between the microarray data and the real-time PCR results was observed (Figure 3). We also measured the basal expression levels of selected genes including several olfactory receptors in the epididymal fat tissues of ND- or HFD-fed mice, using real-time PCR. The results indicated that the basal expression levels of highly ranked olfactory genes (Olfr1181, Olfr513, Olfr960, and Olfr1245) were comparable to those of top five genes (Gli2, Gucy2c, Atp8b3, and Tmem46) identified by MNI analysis at week 4 in the epididymal adipose tissue (Figure S1).

**Table 1:** List of top 5 genes identified by the MNI analysis at each time point in the epididymal fat tissue of HFD-induced obese mice.

	Rank		Gene accession No.	Gene symbol	Description	Function	Fold change	
***Epididymal fat tissue***								
2 week	1	NM_023173		Dusp12	Dual specificity phosphatase 12	Insulin resistance		2.52
	2	NM_172461		Nek11	NIMA (never in mitosis gene a)-related expressed kinase 11	Cancer		0.43
	3	NM_145489		AI661453	Expressed sequence AI661453	Unknown		0.23
	4	NM_177078		Adrbk2	Adrenergic receptor kinase, beta 2	Bipolar disorder		2.73
	5	NM_013679		Svs6	Seminal vesicle secretory protein 6	Unknown		0.77
4 week	1	XM_136212		Gli2	GLI-Kruppel family member GLI2	Cancer		2.73
	2	NM_145067		Gucy2c	Guanylate cyclase 2c	Cancer		0.34
	3	NM_001011816		Olfr1181	Olfactory receptor 1181	Olfactory transduction		5.47
	4	NM_026094		Atp8b3	ATPase, Class I, type 8B, member 3	ATP binding		3.25
	5	NM_145463		Tmem46	Transmembrane protein 46	Cancer		0.55
8 week	1	NM_199155		Tas2r110	Taste receptor, type 2, member 110	Sensory perception of taste		2.81
	2	AK138164		Cntn5	Contactin 5	Cell adhesion		0.33
	3	NM_152220		Stx3	Syntaxin 3	Arachidonic acid binding		1.81
	4	NM_178924		Upk1b	Uroplakin 1B	Epithelial cell differentiation		1.79
	5	NM_028622		Lce1c	Late cornified envelope 1C	Unknown		0.32
12 week	1	NM_139270		Pthr2	Parathyroid hormone receptor 2	Parathyroid hormone receptor activity		2.39
	2	NM_172853		Cdh7	Cadherin 7, type 2	Calcium ion binding		2.42
	3	NM_008586		Mep1b	Meprin 1 beta	Cancer		0.33
	4	NM_011836		Lamc3	Laminin gamma 3	Cell adhesion		0.49
	5	NM_145463		Tmem46	Transmembrane protein 46	Cancer		0.59

**Table 2:** List of top 5 genes identified by the MNI analysis at each time point in the subcutaneous fat tissue of HFD-induced obese mice.

	Rank	Gene accession No.	Gene symbol	Description	Function			Fold change
***Subcutaneous fat tissue***								
2 week	1	NM_025779	Ccdc109b	Coiled-coil domain containing 109B		Cancer	0.54	
	2	NM_001025438	Camk2d	Calcium/calmodulin-dependent protein kinase II, delta		Calmodulin binding	0.65	
	3	AB211064	L1td1	LINE-1 type transposase domain containing 1		Unknown	2.61	
	4	NM_026345	Mansc1	MANSC domain containing 1		Unknown	3.03	
	5	NM_207566	Olfr1173	Olfactory receptor 1173		Olfactory transduction	1.97	
4 week	1	NM_008529	Ly6e	Lymphocyte antigen 6 complex, locus E		Adrenal gland development	1.31	
	2	NM_146524	Olfr855	Olfactory receptor 855		Olfactory transduction	1.57	
	3	NM_018744	Sema6a	Sema domain, transmembrane domain (TM), and cytoplasmic domain, (semaphorin) 6A		Nervous system development	0.73	
	4	AB211064	L1td1	LINE-1 type transposase domain containing 1		Unknown	3.13	
	5	NM_183015	Ccnb3	Cyclin B3		Cell cycle	2.58	
8 week	1	NM_153111	Fev	FEV (ETS oncogene family)		Nervous system development	0.39	
	2	AB211064	L1td1	LINE-1 type transposase domain containing 1		Unknown	2.8	
	3	NM_147018	Olfr1056	Olfactory receptor 1056		Olfactory transduction	0.73	
	4	NM_008999	Rab23	RAB23, member RAS oncogene family		Cancer	0.56	
	5	NM_080644	Cacng5	Calcium channel, voltage-dependent, gamma subunit 5		Calcium ion transport	0.36	
12 week	1	NM_001024852	Auts2	Autism susceptibility candidate 2		Mental retardation	0.53	
	2	NM_178046	Svil	Supervillin		Unknown	0.57	
	3	NM_018764	Pcdh7	Protocadherin 7		Cell adhesion	1.82	
	4	NM_146604	Olfr716	Olfactory receptor 716		Olfactory transduction	3.08	
	5	BC089489	4930474M22Rik	RIKEN cDNA 4930474M22 gene		Unknown	1.9	

**Table 3:** List of top 5 genes identified by the MNI analysis at each time point in the gastrocnemius muscle of HFD-induced obese mice.

	Rank	Gene accession No.	Gene symbol	Description	Function	Fold change
***Gastrocnemius muscle***						
2 week	1	NM_019574	Patz1	POZ (BTB) and AT hook containing zinc finger 1	Cancer	1.8
	2	NM_020610	Nrip3	Nuclear receptor interacting protein 3	Inflammation	0.42
	3	NM_024124	Hdac9	Histone deacetylase 9	Cancer	0.55
	4	XM_975536	Armc4	Armadillo repeat containing 4	Unknown	0.54
	5	NM_139226	Onecut3	One cut domain, family member 3	DNA binding	1.34
4 week	1	NM_153589	Tmem16b	Transmembrane protein 16B	Olfactory transduction	0.74
	2	NM_175540	Eda2r	Ectodysplasin A2 isoform receptor	Alopecia	0.88
	3	NM_008355	Il13	Interleukin 13	Inflammation	1.57
	4	NM_010608	Kcnk3	Potassium channel, subfamily K, member 3	Ion transport	1.22
	5	XM_129809	Ogfrl1	Opioid growth factor receptor-like 1	Unknown	0.7
8 week	1	NM_024124	Hdac9	Histone deacetylase 9	Cancer	0.6
	2	NM_011990	Slc7a11	Solute carrier family 7	Amino acid transport	0.7
	3	NM_175420	9330176C04Rik	RIKEN cDNA 9330176C04 gene	Unknown	2.17
	4	NM_016961	Mapk9	Mitogen activated protein kinase 9	Insulin resistance	0.59
	5	NM_027462	Wars2	Tryptophanyl tRNA synthetase 2 (mitochondrial)	Vasculogenesis	0.56
12 week	1	NM_008114	Gfi1	Growth factor independent 1B	Hematopoiesis	0.74
	2	NM_145435	Pyy	Peptide YY	Insulin resistance	1.49
	3	NM_138648	Olr1	Oxidized low density lipoprotein (lectin-like) receptor 1	Inflammation	0.33
	4	NM_177861	Tmem67	Transmembrane protein 67	Mental retardation	0.54
	5	XM_887155	Igsf10	Immunoglobulin superfamily, member 10	Unknown	0.42

### Functional analysis of the highly ranked genetic mediators

We next focused on the GO-annotated pathways that were significantly overrepresented among the highly ranked genetic mediators. For our analysis, we subjected the 100 highest ranked genes identified by MNI analysis in the epididymal and subcutaneous fat tissue and gastrocnemius muscle of mice with diet-induced obesity to pathway analysis based on the GO biological process annotations (Tables 4, 5, 6). We found that the olfactory transduction was highly enriched in the epididymal and subcutaneous fat tissue and gastrocnemius muscle of the HFD-fed mice compared to the ND-fed mice at all time points. Even the second representative functional theme of epididymal fat was related to cancer at all time points. The pathways thought to be associated with obesity progression in the epididymal fat as per the MNI analysis included Wnt signaling pathway, melanogenesis, chemokine signaling pathway, focal adhesion, MAPK signaling pathway, purine metabolism, regulation of actin cytoskeleton, neuroactive ligand-receptor interaction, and extracellular matrix (ECM)-receptor interaction. In the subcutaneous fat, other pathways identified by the MNI analysis for obesity progression included calcium signaling pathway, gonadotropin-releasing hormone (GnRH) signaling pathway, axon guidance, cell cycle, and tyrosine metabolism. In the gastrocnemius muscle, besides the olfactory transduction mentioned above, the over-represented groups identified according to GO biological processes for obesity progression were those involved in the various cellular processes such as neuroactive ligand-receptor interaction, cytokine-cytokine receptor interaction, pathways associated with cancer, insulin signaling pathway, pathways associated with colorectal cancer, adipocytokine signaling pathway, type II diabetes mellitus, and cell adhesion molecules.

**Table 4:** The enriched pathways among top 100 genetic mediators identified by the MNI analysis at each time point in the epididymal fat tissue of HFD-induced obese mice.

		GO ontology	Ranked pathway genes (rank)
***Epididymal fat tissue***			
2 week	Olfactory transduction		Adrbk2 (4), Olfr513 (6), Olfr433 (15), Camk2g (28), Olfr1245 (29), Olfr1143 (36), Olfr996 (55), Olfr960 (57), Arrb2 (79)
	Wnt signaling pathway		Camk2g (28), Rhoa (31), Wnt10a (95)
	Melanogenesis		Camk2g (28), Adcy5 (80), Wnt10a (95)
	Chemokine signaling pathway		Rhoa (31), Arrb2 (79), Adcy5 (80)
	Focal adhesion		Rhoa (31), Lamc3 (44), Bcl2 (77)
	Pathways in cancer		Rhoa (31), Bcl2 (77), Wnt10a (95)
4 week	Olfactory transduction		Camk2g (17), Olfr513 (27), Olfr960 (31), Olfr1245 (43), Arrb2 (55)
	Pathways in cancer		Gli2 (1), Lamc3 (9), Bcl2 (58), Fgf5 (65)
	MAPK signaling pathway		Arrb2 (55), Fgf5 (65), Mapkapk5 (75)
	Purine metabolism		Gucy2c (2), Nme7 (47), Cant1 (85)
8 week	Olfactory transduction		Olfr536 (11), Olfr513 (31), Olfr654 (37), Camk2g (74), Olfr652 (99)
	Focal adhesion		Flnc(16), Bcl2 (64), Col6a2 (83), Rhoa (86), Mylk (96),
	Regulation of actin cytoskeleton		Fgf5 (26), Rhoa (86), Mylk (96)
	Pathways in cancer		Fgf5 (26), Bcl2 (64), Rhoa (86)
12 week	Olfactory transduction		Olfr16 (6), Camk2g (10), Olfr715 (24), Olfr1245 (32), Olfr536 (36), Olfr1143 (52)
	Neuroactive ligand-receptor interaction		Pth2r (1), Agtrl1 (16), Vipr2 (27), P2rx6 (74)
	Focal adhesion		Lamc3 (4), Bcl2 (75), Col6a2 (76), Rhoa (88)
	ECM-receptor interaction		Lamc3 (4), Cd44 (59), Col6a2 (76)
	Pathways in cancer		Lamc3 (4), Bcl2 (75), Rhoa (88)

**Table 5:** The enriched pathways among top 100 genetic mediators identified by the MNI analysis at each time point in the subcutaneous fat tissue of HFD-induced obese mice.

	GO ontology	Ranked pathway genes (rank)
***Subcutaneous fat tissue***		
2 week	Olfactory transduction	Camk2b (2), Olfr1173 (5), Olfr823 (9), Guca1a (41), Olfr411 (49), Olfr1408 (51), Olfr875 (64)
	Calcium signaling pathway	Camk2b (2), Cacna1d (15), Htr4 (45), Ryr1 (96)
	GnRH signaling pathway	Camk2b (2), Cacna1d (15), Cga (48)
4 week	Olfactory transduction	Olfr855 (2), Olfr888 (13), Olfr305 (36), Olfr1173 (49), Olfr395 (52), Olfr411 (55), Olfr823 (83), Olfr1409 (89)
	Axon guidance	Sema6a (3), Abl1 (23)
	Cell cycle	Ccnb3 (5), Abl1 (23)
	Regulation of actin cytoskeleton	Vav3 (9), Ssh2 (25)
	Tyrosine metabolism	Fah (26), Aoc3 (27)
	MAPK signaling pathway	Cacna1d (28), Hspa1a (44)
8 week	Olfactory transduction	Olfr1056 (3), Olfr960 (15), Olfr1408 (26), Olfr855 (35), Olfr609 (42), Olfr1173 (49), Olfr411 (71), Olfr205 (91), Olfr1121 (94)
	Focal adhesion	Shc4 (63), Diap1 (77), Lamb2 (82), Vav3 (83)
12 week	Olfactory transduction	Olfr716 (4), Olfr45 (16), Olfr960 (19), Olfr1173 (32), Olfr411 (34), Olfr1408 (43), Olfr855 (45), Olfr875 (96)
	GnRH signaling pathway	Cacna1d (29), Cga (58)
	Chemokine signaling pathway	Vav3 (33), Gng3 (79), Shc4 (84)

**Table 6:** The enriched pathways among top 100 genetic mediators identified by the MNI analysis at each time point in the gastrocnemius muscle of HFD-induced obese mice.

	GO ontology	Ranked pathway genes (rank)
***Gastrocnemius muscle***		
2 week	Olfactory transduction	Clca3 (6), Olfr739 (14), Olfr488 (40), Olfr474 (55)
4 week	Olfactory transduction	Olfr800 (28), Olfr978 (33), Olfr474 (49), Olfr488 (73)
	Neuroactive ligand-receptor interaction	Oprl1 (30), Mc1r (78), Tbxa2r (89), Tspo (97)
	Cytokine-cytokine receptor interaction	Eda2r (2), Il13 (3), Cx3cl1 (91)
	Pathways in cancer	Dcc (10), Amn (71), Mlh1 (85)
8 week	Olfactory transduction	Clca3 (9), Olfr739 (48), Olfr1305 (64), Olfr1395 (80), Olfr140 (81), Olfr63 (85), Olfr689 (86)
	Pathways in cancer	Mapk9 (4), Dcc (15), Mapk10 (78), Grb2 (87), Mitf (89)
	Insulin signaling pathway	Mapk9 (4), Irs3 (40), Mapk10 (78), Grb2 (87)
	Colorectal cancer	Mapk9 (4), Dcc (15), Mapk10 (78), Grb2 (87)
	Adipocytokine signaling pathway Type II diabetes mellitus	Mapk9 (4), Irs3 (40), Mapk10 (78)
12 week	Olfactory transduction	Olfr689 (22), Olfr488 (24), Olfr347 (38), Olfr1204 (69)
	Cell adhesion molecules (CAMs)	Cd22 (23), Nlgn3 (44), Ptprm (62)
	Cytokine-cytokine receptor interaction	Eda2r (43), Ppbp (53), Cx3cl1 (57)

### Representative time-course profile clusters

**Figure 4:**
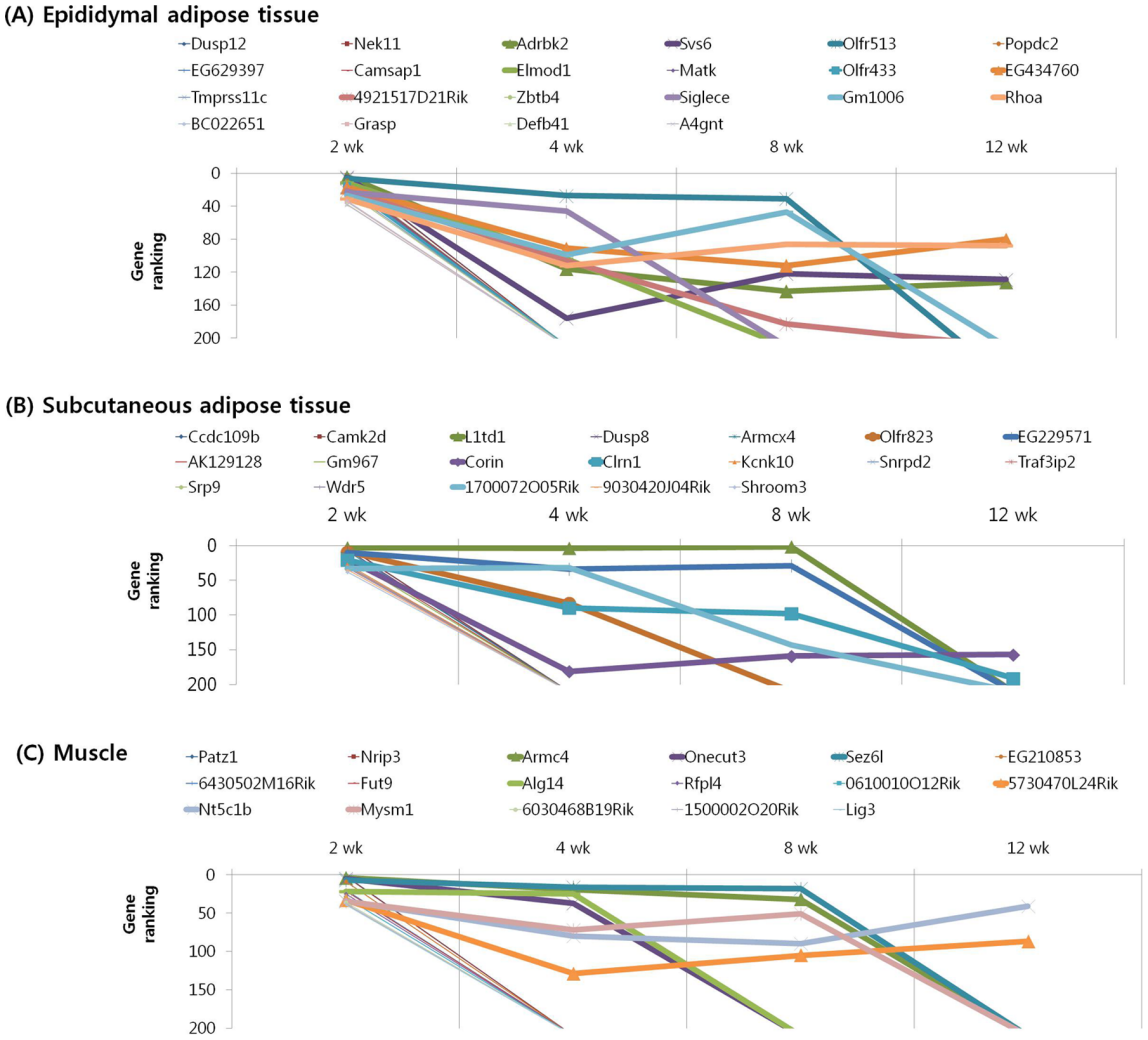
Results of MNI analysis at week 2. Genes that exhibited decreasing ranking across time points as revealed by the MNI analysis, with a peak at 2 week in the peripheral tissues of mice. (A) Epididymal adipose tissue. (B) Subcutaneous adipose tissue. (C) Muscle.

**Figure 5:**
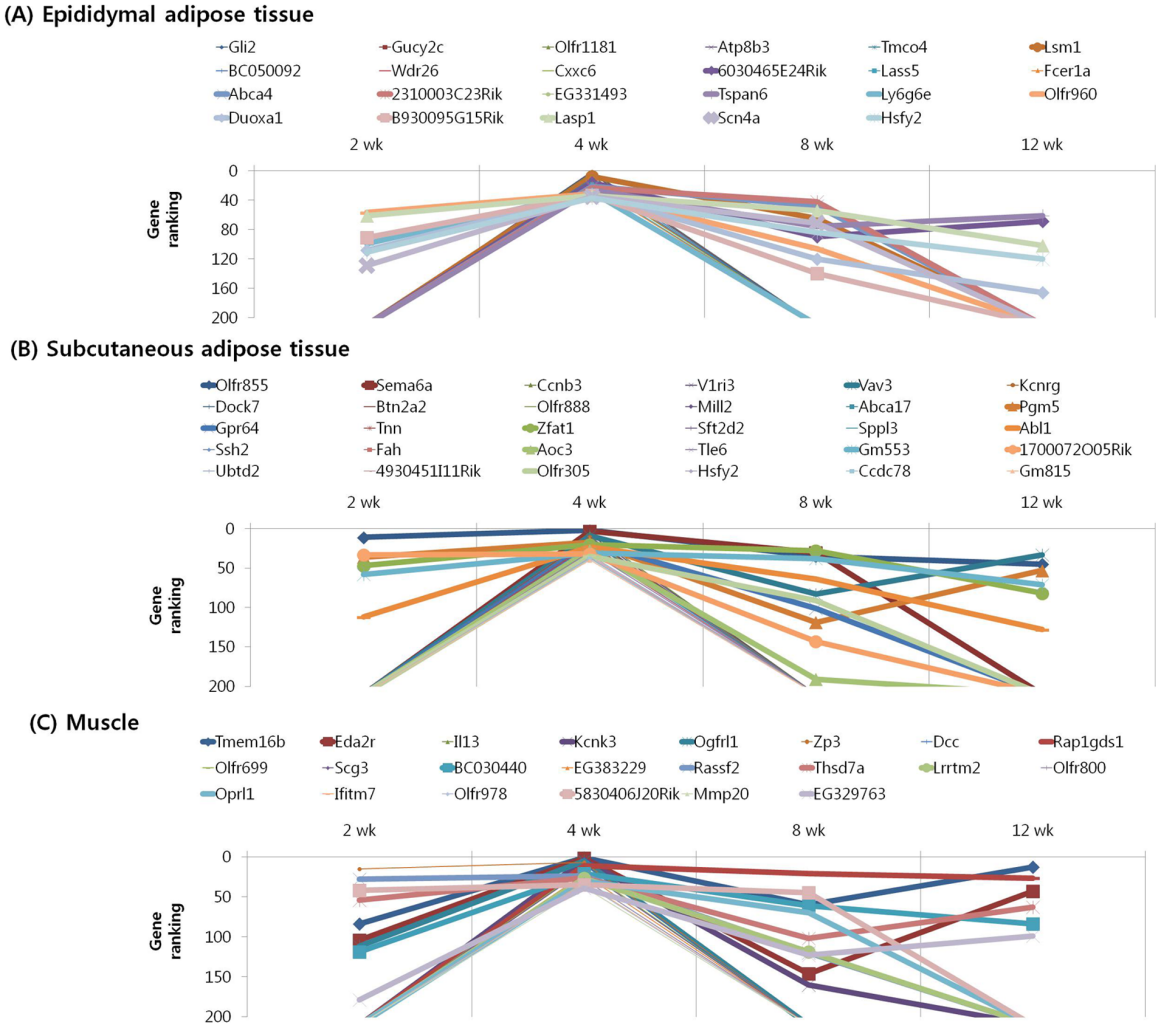
Results of MNI analysis at week 4. Genes that exhibited the highest rank at the intermediate time point of 4 week as revealed by the MNI analysis in the peripheral tissues of mice. (A) Epididymal adipose tissue. (B) Subcutaneous adipose tissue. (C) Muscle.

**Figure 6:**
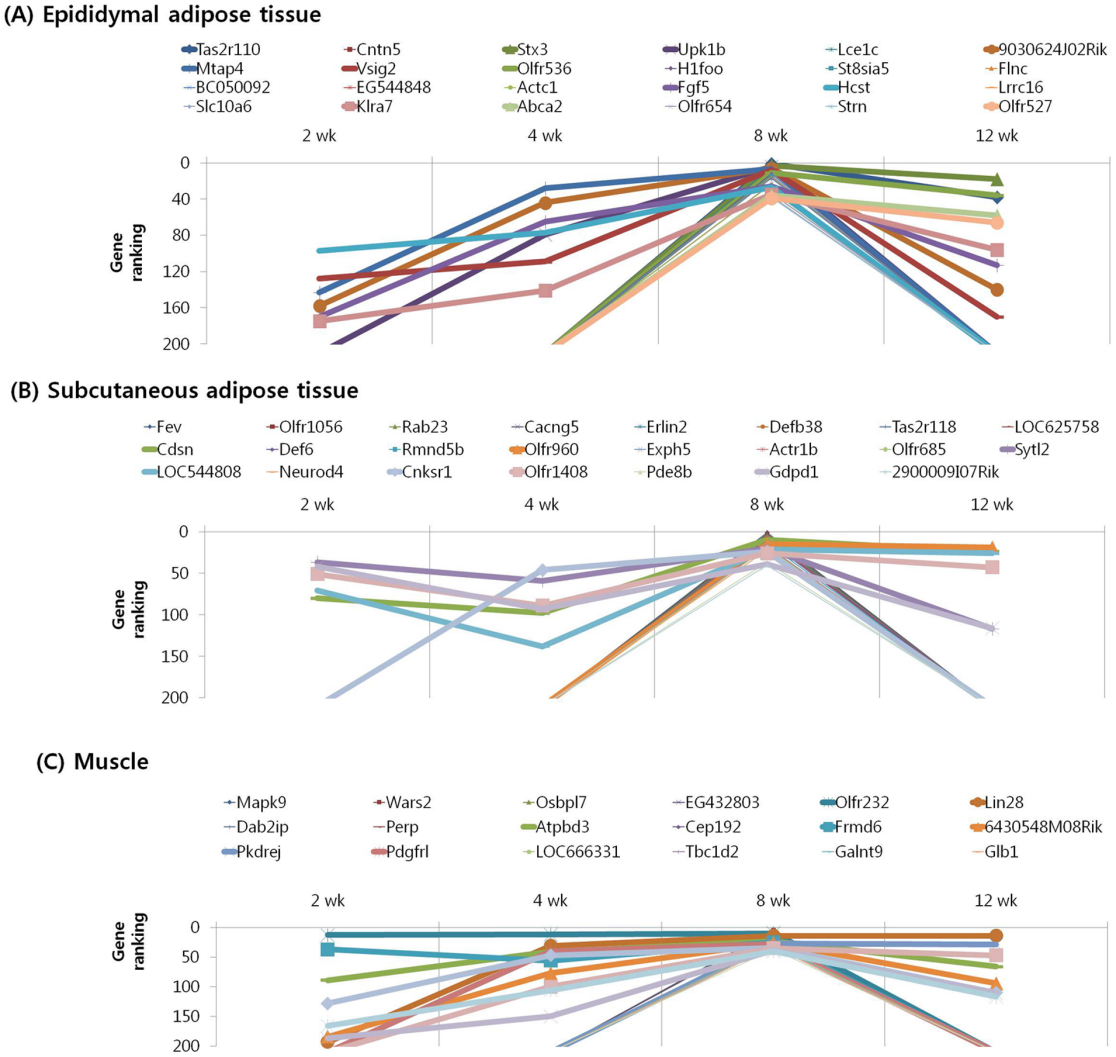
Results of MNI analysis at week 8. Genes that exhibited the highest rank at the intermediate time point of 8 week as revealed by the MNI analysis in the peripheral tissues of mice. (A) Epididymal adipose tissue. (B) Subcutaneous adipose tissue. (C) Muscle.

**Figure 7:**
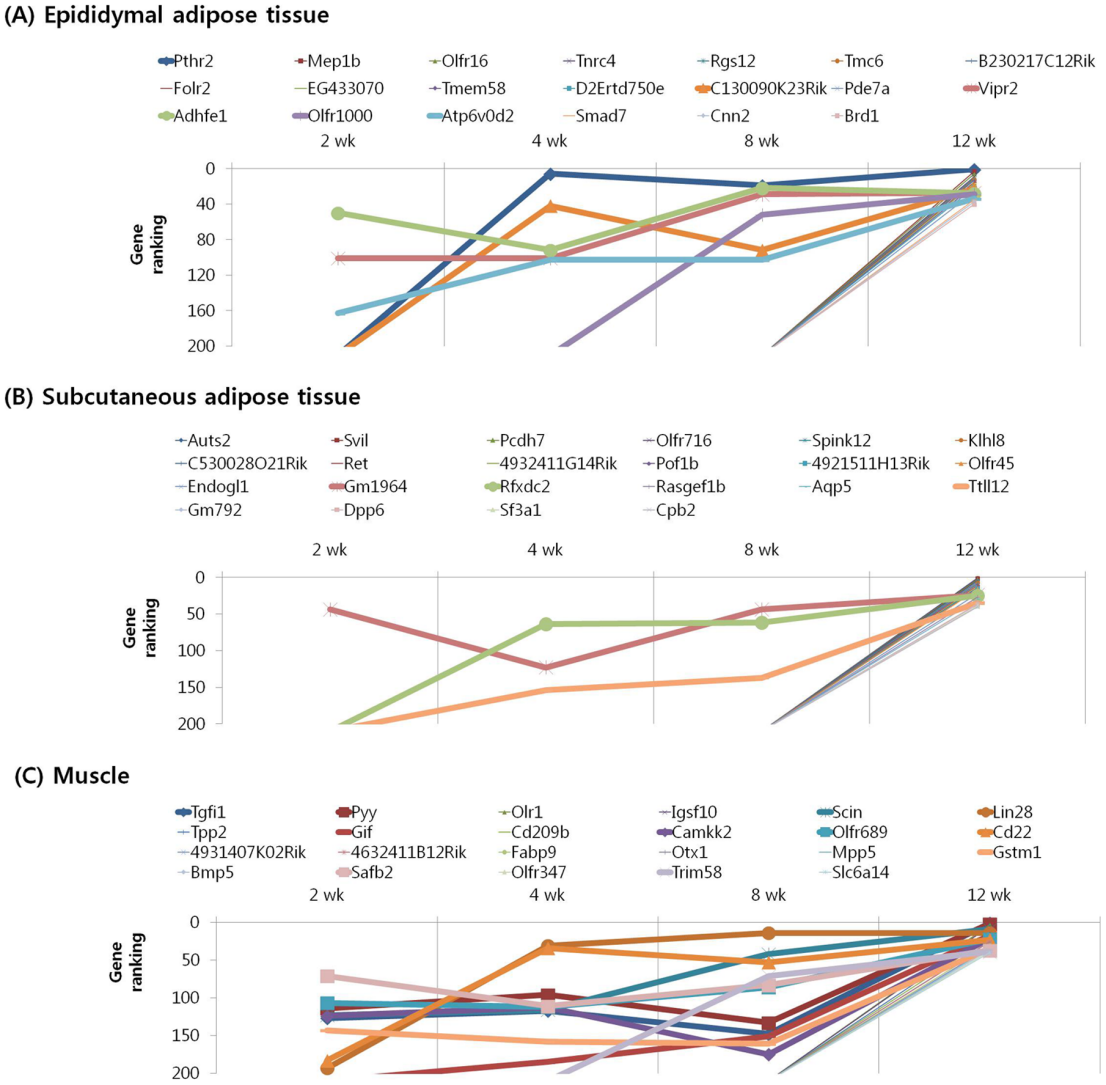
Results of MNI analysis at week 12. Genes that exhibited increasing ranking across time points as revealed by the MNI analysis, with a peak at 12 week in the peripheral tissues of mice. (A) Epididymal adipose tissue. (B) Subcutaneous adipose tissue. (C) Muscle.

**Figure 8:**
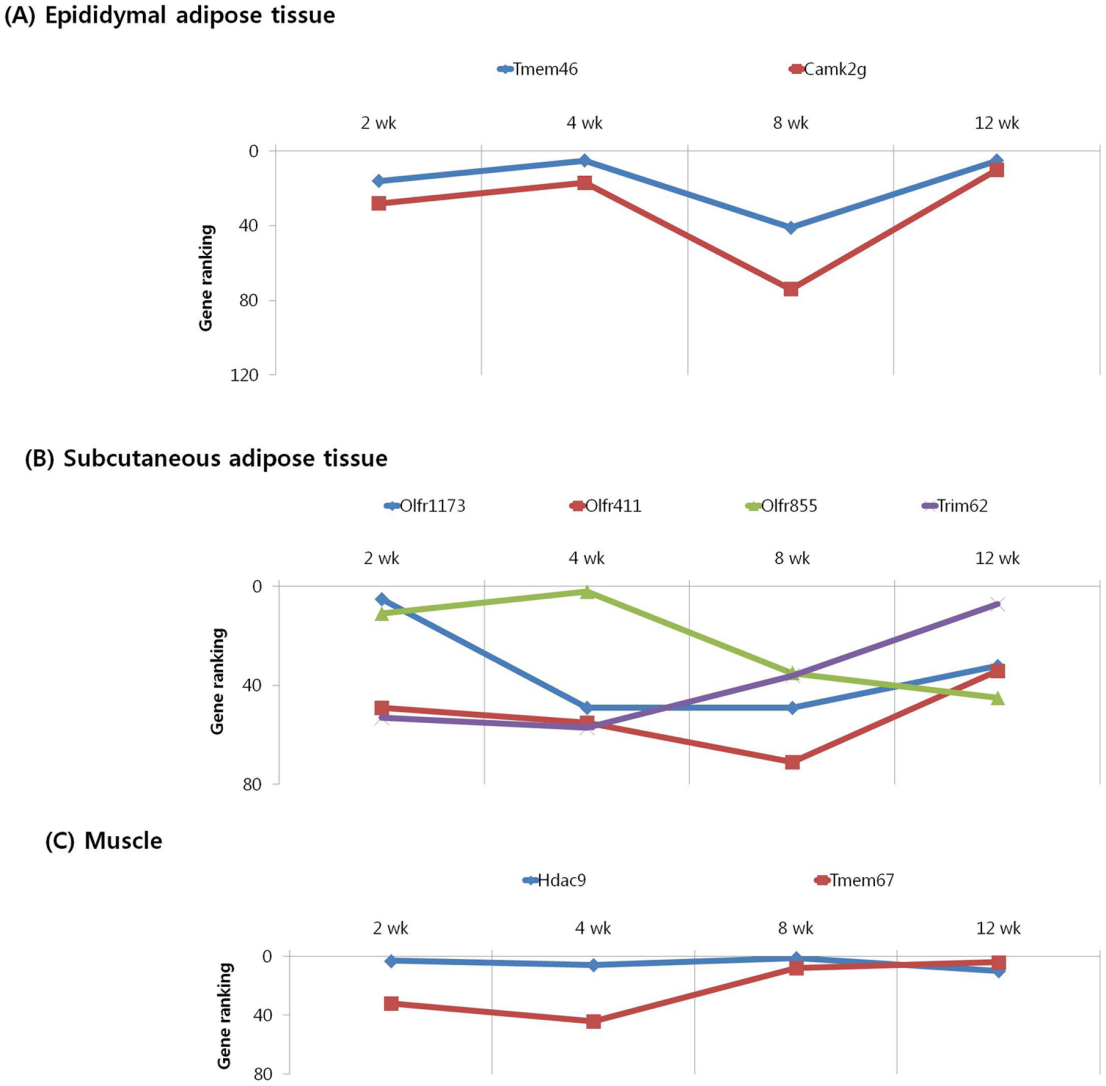
Results of MNI analysis throughout the time course. Genes that exhibited a constant high MNI ranking throughout the time course as revealed by the MNI analysis in the peripheral tissues of mice. (A) Epididymal adipose tissue. (B) Subcutaneous adipose tissue. (C) Muscle.

We subjected the 40 highest ranked genes identified by MNI analysis at each time point in the epididymal and subcutaneous fat tissues and gastrocnemius muscle of mice with diet-induced obesity to clustering based on their temporal pattern. Figure 4 shows genes that were observed to have decreasing ranking across time point, with a peak at 2 week. Biological processes controlled by genes in this cluster included regulation of insulin resistance (EA: Dusp12), cancer (EA: Nek11, A4gnt; SA: Srp9), inflammation (EA: Siglece; M: Nrip3, Sez6l), and olfactory transduction (EA: Olfr 513, 433; SA: Olfr 823) (Tables 7, 8, 9). The genes shown in Figures 5 and 6 exhibited the highest rank at the intermediate time points of 4 and 8 weeks, respectively. For both clusters, the majority of genes in this category were associated with cancer (EA: Gli2, Gucy2c, Lsm1, Duoxa1, Lasp1; SA: Vav3, Kcnrg, Tle6, Rab23; M: Dcc, Rassf2, Perp, Pdgfr1), inflammation (SA: Btn2a2, Def6; M: ll13, Rap1gds1), insulin resistance (SA: Neurod4; M: Mapk9), and olfactory transduction (EA: Olfr 1181, 960, 536, 654, 527; SA: Olfr 855, 888, 305, 1056, 960, 685, 1048; M: Olfr 699, 800, 978, 232, 872) (Tables 10, 11, 12, 13, 14, 15). Figure 7 shows genes that exhibited increasing ranking across all time points, with a peak at 12 week. Biological processes for genes in this cluster included regulation of adipogenesis (EA: Smad7, Adhfe1), food intake (M: Pyy), inflammation (EA: Folr2, Pde7a, Vipr2; SA: Rfxdc2, Aqp5, Cpb2), cancer (M: Lin28, Gstm1, Safb2), and olfactory transduction (EA: Olfr 16, 1000; SA: Olfr 716, 45; M: Olfr 689, 347) (Tables 16, 17, 18). The genes shown in Figure 8 exhibited a constant high MNI ranking throughout the time course. This cluster contained genes related to cancer (EA: Tmem46; SA: Trim62; M: Hdac9), insulin resistance (EA: Camk2g), hepatic fibrosis (M: Tmem67), and olfactory transduction (SA: Olfr 1173, 411, 855) (Table 19).

**Table 7:** List of genes that exhibited decreasing ranking across time point as revealed by the MNI analysis, with a peak at 2 week in the epididymal adipose tissue of mice.

Gene accession No.	Gene symbol	Description	Fold change (HFD/ND)			
			2wk	4wk	8wk	12wk
**Epididymal adipose tissue**						
***Insulin resistance***						
NM_023173	Dusp12	Dual specificity phosphatase 12	2.5	1.8	2.1	1.7
***Inflammation***						
NM_031181	Siglece	Sialic acid binding Ig-like lectin E	2.1	1.9	1.7	1.7
***Cancer***						
NM_172461	Nek11	NIMA (never in mitosis gene a)-related expressed kinase 11	0.4	0.5	0.6	0.6
XM_286168	A4gnt	Alpha-1,4-N-acetylglucosaminyltransferase	2.0	1.7	1.6	1.5
***Olfactory transduction***						
NM_146723	Olfr513	Olfactory receptor 513	0.3	0.3	0.3	0.4
NM_146717	Olfr433	Olfactory receptor 433	2.1	2.0	1.9	1.5
***Others***						
NM_022318	Popdc2	Popeye domain containing 2	2.6	2.0	1.8	1.9
XM_901428	EG629397	Predicted gene, EG629397	1.8	1.7	1.5	1.4
XM_899101	Camsap1	Calmodulin regulated spectrin-associated protein 1-like 1	3.4	2.2	2.3	2.6
NM_177769	Elmod1	ELMO domain containing 1	1.7	1.6	1.5	1.4
NM_010768	Matk	Megakaryocyte-associated tyrosine kinase	2.3	1.8	1.7	1.8
NM_177078	Adrbk2	Adrenergic receptor kinase, beta 2	2.7	2.1	2.2	2.1
NM_013679	Svs6	Seminal vesicle secretory protein 6	0.8	0.7	0.7	0.7
XM_486653	EG434760	Predicted gene, EG434760	0.4	0.5	0.4	0.4
NM_001030297	Tmprss11c	Transmembrane protease, serine 11c	1.9	1.5	2.0	1.7
NM_026338	4921517D21Rik	RIKEN cDNA 4921517D21 gene	2.9	2.5	2.8	2.0
NM_133879	Zbtb4	Zinc finger and BTB domain containing 48	0.4	0.2	0.3	0.5
NM_001034875	Gm1006	Gene model 1006, (NCBI)	2.1	2.1	1.7	2.0
NM_016802	Rhoa	Ras homolog gene family, member A	2.1	2.1	1.7	2.0
NM_177887	BC022651	cDNA sequence BC022651	1.8	1.4	1.6	1.5
NM_207670	Grasp	GRIP1 associated protein	1.4	1.2	1.3	1.3
NM_183124	Defb41	Defensin beta 41	0.7	0.8	0.8	0.7

**Table 8:** List of genes that exhibited decreasing ranking across time point as revealed by the MNI analysis, with a peak at 2 week in the subcutaneous adipose tissue of mice.

Gene accession No.	Gene symbol	Description	Fold change (HFD/ND)			
			2wk	4wk	8wk	12wk
**Subcutaneous adipose tissue**						
***Cancer***						
NM_012058	Srp9	Signal recognition particle 9	0.5	0.6	0.7	0.5
***Olfactory transduction***						
NM_146673	Olfr823	Olfactory receptor 823	0.6	0.6	0.7	0.7
***Others***						
NM_025779	Ccdc109b	Coiled-coil domain containing 109B	0.5	0.7	0.7	0.6
NM_001025438	Camk2d	Calcium/calmodulin-dependent protein kinase II, delta	0.8	0.8	0.8	0.8
AB211064	L1td1	LINE-1 type transposase domain containing 1	3.1	2.8	1.9	1.9
NM_008748	Dusp8	Dual specificity phosphatase 8	0.6	0.6	0.8	0.8
XM_905633	Armcx4	Armadillo repeat containing, X-linked 4	1.9	2.0	2.4	2.4
NM_001034860	EG229571	Predicted gene, EG229571	1.6	1.5	1.4	1.4
XM_898433	AK129128	cDNA sequence AK129128	2.3	1.8	1.9	1.4
XM_355152	Gm967	Gene model 967, (NCBI)	0.5	0.6	0.6	0.7
NM_016869	Corin	Corin	0.7	0.7	0.8	0.8
NM_153386	Clrn1	Clarin 1	1.3	1.2	1.3	1.3
NM_029911	Kcnk10	Potassium channel, subfamily K, member 10	0.7	0.7	0.8	0.8
NM_026943	Snrpd2	Small nuclear ribonucleoprotein D2	0.6	0.8	0.7	0.7
NM_134000	Traf3ip2	Traf3 interacting protein 2	1.5	1.3	1.2	1.2
NM_023790	Wdr5	WD repeat domain 54	1.8	1.3	1.3	0.9
XM_987216	1700072O05Rik	RIKEN cDNA 1700072O05 gene	0.6	0.6	0.6	0.7
XM_146632	9030420J04Rik	RIKEN cDNA 9030420J04 gene	2.5	2.1	2.0	1.5
NM_015756	Shroom3	Shroom family member 3	1.9	1.4	1.3	1.5

**Table 9:** List of genes that exhibited decreasing ranking across time point as revealed by the MNI analysis, with a peak at 2 week in the muscle of mice.

Gene accession No.	Gene symbol	Description	Fold change (HFD/ND)			
			2wk	4wk	8wk	12wk
**Muscle**						
***Inflammation***						
NM_020610	Nrip3	Nuclear receptor interacting protein 3	0.4	0.5	0.7	0.5
BC065117	Sez6l	Seizure related 6 homolog like	1.7	1.7	1.7	1.3
***Others***						
NM_019574	Patz1	POZ (BTB) and AT hook containing zinc finger 1	1.8	1.4	1.3	1.5
XM_975536	Armc4	Armadillo repeat containing 4	0.5	0.4	0.5	0.6
NM_139226	Onecut3	One cut domain, family member 3	1.3	1.4	1.3	1.2
NM_177596	EG210853	Predicted gene, EG210853	0.4	0.6	0.7	0.7
NM_175455	6430502M16RIk	RIKEN cDNA 6430502M16 gene	2.3	1.6	1.7	1.7
NM_010243	Fut9	Fucosyltransferase 9	0.8	0.9	0.9	0.9
NM_024178	Alg14	Asparagine-linked glycosylation 14 homolog (yeast)	0.4	0.5	0.6	0.6
NM_138954	Rfpl4	Ret finger protein-like 4	0.6	0.8	0.7	0.7
XM_900215	0610010O12Rik	RIKEN cDNA 0610010O12 gene	1.6	1.3	1.4	1.3
NM_025679	5730470L24Rik	RIKEN cDNA 5730470L24 gene	0.8	0.8	0.8	0.8
NM_027588	Nt5c1b	5′-nucleotidase, cytosolic IB	0.7	0.7	0.7	0.6
NM_177239	Mysm1	Myb-like, SWIRM and MPN domains 1	0.5	0.6	0.5	0.7
XM_126537	6030468B19Rik	RIKEN cDNA 6030468B19 gene	1.6	1.4	1.3	1.2
NM_028047	1500002O20Rik	RIKEN cDNA 1500002O20 gene	1.3	1.2	1.3	1.2
U66058	Lig3	Ligase III, DNA, ATP-dependent	0.8	0.8	0.8	0.8

**Table 10:** List of genes that exhibited the highest ranking at the intermediate time point of 4 week as revealed by the MNI analysis in the epididymal adipose tissue of mice.

Gene accession No.	Gene symbol	Description	Fold change (HFD/ND)			
			2wk	4wk	8wk	12wk
**Epididymal adipose tissue**						
***cancer***						
XM_136212	Gli2	GLI-Kruppel family member GLI2	2.2	2.7	2.4	1.8
NM_145067	Gucy2c	Guanylate cyclase 2c	0.4	0.3	0.5	0.5
NM_138721	Lsm1	U7 snRNP-specific Sm-like protein LSM10	0.3	0.9	0.8	0.6
NM_145395	Duoxa1	Dual oxidase maturation factor 1	1.7	1.7	1.7	1.5
NM_010688	Lasp1	LIM and SH3 protein 1	2.1	2.5	2.8	2.3
***olfacroty transduction***						
NM_001011816	Olfr1181	Olfactory receptor 1181	3.1	5.5	2.9	3.9
NM_146279	Olfr960	Olfactory receptor 960	2.4	2.3	2.1	1.8
***Others***						
NM_026094	Atp8b3	ATPase, Class I, type 8B, member 3	2.5	3.3	2.5	2.1
NM_029857	Tmco4	Transmembrane and coiled-coil domains 4	0.5	0.3	0.6	0.4
NM_181419	BC050092	cDNA sequence BC050092	2.2	3.4	2.8	2.6
NM_145514	Wdr26	WD repeat domain 26	0.4	0.3	0.4	0.4
XM_125673	Cxxc6	CXXC finger 6	2.4	2.9	2.2	1.8
BC019404	6030465E24Rik	RIKEN cDNA 6030465E24 gene	1.4	1.7	1.6	1.6
NM_028015	Lass5	Longevity assurance homolog 5 (S. cerevisiae)	2.4	3.5	2.4	2.3
NM_010184	Fcer1a	Fc receptor, IgE, high affinity I, alpha polypeptide	0.6	0.5	0.7	0.6
NM_007378	Abca4	ATP-binding cassette, sub-family A (ABC1), member 4	1.7	1.9	1.8	1.5
NM_029607	2310003C23Rik	RIKEN cDNA 2310003C23 gene	0.4	0.3	0.4	0.5
NM_001033541	EG331493	Predicted gene, EG331493	1.3	1.5	1.4	1.4
NM_019656	Tspan6	Tetraspanin 6	2.0	2.8	2.4	2.2
NM_027366	Ly6g6e	Lymphocyte antigen 6 complex, locus G6E	1.7	1.5	1.5	1.4
BC096543	B930095G15Rik	RIKEN cDNA B930095G15 gene	3.6	3.8	2.7	2.7
NM_133199	Scn4a	Sodium channel, voltage-gated, type IV, alpha	0.4	0.4	0.5	0.5
NM_027661	Hsfy2	Heat shock transcription factor, Y linked 2	1.4	1.4	1.5	1.3

**Table 11:** List of genes that exhibited the highest ranking at the intermediate time point of 4 week as revealed by the MNI analysis in the subcutaneous adipose tissue of mice.

Gene accession No.	Gene symbol	Description	Fold change (HFD/ND)			
			2wk	4wk	8wk	12wk
**Subcutaneous adipose tissue**						
***Inflammation***						
NM_175938	Btn2a2	Butyrophilin, subfamily 2, member A2	1.6	2.4	1.8	1.5
***Cancer***						
NM_020505	Vav3	Vav 3 oncogene	1.3	1.6	1.4	1.5
NM_206974	Kcnrg	Potassium channel regulator	1.3	1.7	1.5	1.3
NM_053254	Tle6	Transducin-like enhancer of split 6, homolog of drosophila E(spl)	0.7	0.6	0.8	0.7
***olfactory transduction***						
NM_146524	Olfr855	Olfactory receptor 855	1.7	1.6	2.0	1.6
NM_146424	Olfr888	Olfactory receptor 888	0.6	0.5	0.6	0.7
NM_146616	Olfr305	Olfactory receptor 305	0.7	0.6	0.6	0.7
***Others***						
NM_018744	Sema6a	Sema domain, transmembrane domain (TM), and cytoplasmic domain, (semaphorin) 6A	0.8	0.7	0.7	0.8
NM_183015	Ccnb3	Cyclin B3	1.8	2.6	1.5	1.8
NM_134220	V1ri3	Vomeronasal 1 receptor, I3	1.6	2.3	1.6	1.7
NM_026082	Dock7	Dedicator of cytokinesis 7	1.6	2.2	1.5	1.4
NM_153761	Mill2	MHC I like leukocyte 2	0.7	0.6	0.7	0.8
NM_001031621	Abca17	ATP-binding cassette, sub-family A (ABC1), member 17	1.3	1.7	1.4	1.4
NM_175013	Pgm5	Phosphoglucomutase 5	1.5	1.5	1.5	1.3
NM_178712	Gpr64	G protein-coupled receptor 64	1.3	1.6	1.6	1.4
NM_177839	Tnn	Tenascin N	0.8	0.7	0.8	0.9
NM_198644	Zfat1	ZFAT zinc finger 1	2.1	2.7	2.1	2.0
NM_145512	Sft2d2	SFT2 domain containing 2	1.2	1.5	1.4	1.3
NM_029012	Sppl3	Signal peptide peptidase 3	0.7	0.5	0.7	0.7
NM_009594	Abl1	v-abl Abelson murine leukemia oncogene 1	0.7	0.7	0.7	0.8
NM_177710	Ssh2	Slingshot homolog 2 (Drosophila)	0.7	0.6	0.8	0.7
NM_010176	Fah	Fumarylacetoacetate hydrolase	0.7	0.7	0.8	0.8
NM_009675	Aoc3	Amine oxidase, copper containing 3	1.2	1.4	1.3	1.3
XM_149023	Gm553	Gene model 553, (NCBI)	2.0	1.9	1.8	1.6
XM_987216	1700072O05Rik	RIKEN cDNA 1700072O05 gene	0.6	0.6	0.6	0.7
NM_173784	Ubtd2	Ubiquitin domain containing 2	1.6	2.0	1.6	1.4
NM_183131	4930451I11Rik	RIKEN cDNA 4930451I11 gene	1.8	2.2	1.8	1.4
NM_027661	Hsfy2	Heat shock transcription factor, Y linked 2	1.9	3.0	0.5	0.9
XM_354998	Ccdc78	Coiled-coil domain containing 78	1.2	1.5	1.3	1.2
NM_001033407	Gm815	Gene model 815, (NCBI)	0.7	0.5	0.7	0.7

**Table 12:** List of genes that exhibited the highest ranking at the intermediate time point of 4 week as revealed by the MNI analysis in the muscle of mice.

Gene accession No.	Gene symbol	Description	Fold change (HFD/ND)			
			2wk	4wk	8wk	12wk
**Muscle**						
***Inflammation***						
NM_008355	Il13	Interleukin 13	1.3	1.6	1.3	1.3
NM_145544	Rap1gds1	RAP1, GTP-GDP dissociation stimulator 1	1.3	1.5	1.5	1.4
***Cancer***						
NM_007831	Dcc	Deleted in colorectal carcinoma	0.8	0.6	0.6	0.7
NM_175445	Rassf2	Ras association (RalGDS/AF-6) domain family 2	0.6	0.5	0.7	0.7
***olfactory transduction***						
NM_153589	Tmem16b	Transmembrane protein 16B	0.7	0.7	0.7	0.7
NM_001011862	Olfr699	Olfactory receptor 699	0.5	0.4	0.6	0.7
NM_146548	Olfr800	Olfactory receptor 800	0.7	0.5	0.6	0.6
NM_147105	Olfr978	Olfactory receptor 978	0.7	0.5	0.5	0.7
***Others***						
NM_175540	Eda2r	Ectodysplasin A2 isoform receptor	0.8	0.9	0.8	0.9
NM_010608	Kcnk3	Potassium channel, subfamily K, member 3	1.1	1.2	1.2	1.2
XM_129809	Ogfrl1	Opioid growth factor receptor-like 1	0.8	0.7	0.8	0.8
NM_011776	Zp3	Zona pellucida glycoprotein 3	1.8	2.0	1.5	1.4
NM_009130	Scg3	Secretogranin III	0.6	0.5	0.7	0.8
NM_173732	BC030440	cDNA sequence BC030440	1.3	1.2	1.2	1.2
XM_356935	EG383229	Predicted gene, EG383229	1.3	1.5	1.2	1.3
XM_992003	Thsd7a	Thrombospondin, type I, domain containing 7A	1.5	1.3	1.4	1.4
NM_178005	Lrrtm2	Leucine rich repeat transmembrane neuronal 2	0.8	0.7	0.7	0.8
NM_011012	Oprl1	Opioid receptor-like 1	1.3	1.6	1.5	1.4
NM_028968	Ifitm7	Interferon induced transmembrane protein 7	0.6	0.4	0.6	0.6
NM_175204	5830406J20Rik	RIKEN cDNA 5830406J20 gene	1.6	1.9	1.7	1.5
NM_013903	Mmp20	Matrix metallopeptidase 20 (enamelysin)	0.7	0.7	0.8	0.8
NM_177860	EG329763	Predicted gene, EG329763	0.8	0.7	0.7	0.7

**Table 13:** List of genes that exhibited the highest ranking at the intermediate time point of 8 week as revealed by the MNI analysis in the epididymal adipose tissue of mice.

Gene accession No.	Gene symbol	Description	Fold change (HFD/ND)			
			2wk	4wk	8wk	12wk
**Epididymal adipose tissue**						
***Olfactory transduction***						
NM_146520	Olfr536	Olfactory receptor 536	0.6	0.6	0.5	0.5
NM_146379	Olfr654	Olfactory receptor 654	1.9	2.4	2.8	1.9
NM_001011776	Olfr527	Olfactory receptor 527	1.7	1.5	1.8	1.8
***Others***						
NM_199155	Tas2r110	Taste receptor, type 2, member 110	1.9	2.5	2.8	2.8
AK138164	Cntn5	Contactin 5	0.4	0.4	0.3	0.5
NM_152220	Stx3	Syntaxin 3	1.8	1.4	1.8	1.8
NM_178924	Upk1b	Uroplakin 1B	1.4	1.8	1.8	1.7
NM_028622	Lce1c	Late cornified envelope 1C	0.5	0.4	0.3	0.5
NM_027815	9030624J02Rik	RIKEN cDNA 9030624J02 gene	0.7	0.7	0.8	0.7
NM_008633	Mtap4	Microtubule-associated protein 4	1.4	1.4	1.4	1.2
NM_020518	Vsig2	V-set and immunoglobulin domain containing 2	2.0	1.8	2.1	1.8
NM_138311	H1foo	H1 histone family, member O, oocyte-specific	0.6	0.6	0.5	0.5
NM_153124	St8sia5	ST8 alpha-N-acetyl-neuraminide alpha-2,8-sialyltransferase 5	0.8	0.7	0.7	0.7
XM_898823	Flnc	Filamin C, gamma (actin binding protein 280)	0.5	0.5	0.4	0.4
NM_181419	BC050092	cDNA sequence BC050092	2.2	3.4	2.8	2.6
XM_001004783	EG544848	Predicted gene, EG544848	0.5	0.6	0.5	0.5
NM_009608	Actc1	Actin, alpha, cardiac	1.8	2.3	2.7	2.3
NM_010203	Fgf5	Fibroblast growth factor 5	0.5	0.5	0.5	0.5
NM_011827	Hcst	Hematopoietic cell signal transducer	2.0	2.2	2.0	1.7
NM_026825	Lrrc16	Leucine rich repeat containing 16	1.4	1.5	1.7	1.5
NM_029415	Slc10a6	Solute carrier family 10 (sodium/bile acid cotransporter family), member 6	0.5	0.4	0.3	0.5
U10093	Klra7	Killer cell lectin-like receptor, subfamily A, member 7	1.2	1.2	1.2	1.2
NM_007379	Abca2	ATP-binding cassette, sub-family A (ABC1), member 2	0.5	0.6	0.5	0.4
NM_001039878	Strn	Striatin, calmodulin binding protein 4	0.6	0.6	0.5	0.6

**Table 14:** List of genes that exhibited the highest ranking at the intermediate time point of 8 week as revealed by the MNI analysis in the subcutaneous adipose tissue of mice.

Gene accession No.	Gene symbol	Description	Fold change (HFD/ND)			
			2wk	4wk	8wk	12wk
**Subcutaneous adipose tissue**						
***Insulin resistance***						
NM_007501	Neurod4	Neurogenic differentiation 4	0.8	0.8	0.6	0.7
***Inflammation***						
NM_027185	Def6	Differentially expressed in FDCP 6	1.9	2.0	3.0	1.7
***Cancer***						
NM_008999	Rab23	RAB23, member RAS oncogene family	0.8	0.8	0.6	0.7
***olfactory transduction***						
NM_147018	Olfr1056	Olfactory receptor 1056	0.8	0.9	0.7	0.8
NM_146279	Olfr960	Olfactory receptor 960	1.4	1.6	1.7	1.9
NM_001011857	Olfr685	Olfactory receptor 685	0.8	0.8	0.7	0.9
NM_146764	Olfr1408	Olfactory receptor 1408	0.6	0.6	0.7	0.7
***Others***						
NM_153111	Fev	FEV (ETS oncogene family)	0.7	0.5	0.4	0.6
NM_080644	Cacng5	Calcium channel, voltage-dependent, gamma subunit 5	0.6	0.6	0.4	0.5
NM_153592	Erlin2	ER lipid raft associated 2	1.3	1.3	1.6	1.3
NM_183036	Defb38	Defensin beta 38	1.6	2.1	2.8	2.0
NM_207022	Tas2r118	Taste receptor, type 2, member 118	2.0	1.6	2.9	2.1
XM_132900	LOC625758	Hypothetical LOC625758	2.0	2.4	2.6	1.5
NM_001008424	Cdsn	Corneodesmosin	0.8	0.7	0.6	0.7
NM_025346	Rmnd5b	Required for meiotic nuclear division 5 homolog B (S. cerevisiae)	1.6	1.9	2.8	2.0
NM_176846	Exph5	Exophilin 5	0.5	0.7	0.5	0.5
NM_146107	Actr1b	ARP1 actin-related protein 1 homolog B (yeast)	0.5	0.6	0.3	0.6
XM_986681	Sytl2	Synaptotagmin-like 2	1.3	1.5	1.3	1.4
XM_618920	LOC544808	Hypothetical LOC544808	1.7	1.6	1.7	1.9
XM_110525	Cnksr1	Connector enhancer of kinase suppressor of Ras 1	1.3	1.5	1.4	1.3
NM_172263	Pde8b	Phosphodiesterase 8B	0.8	0.7	0.6	0.8
NM_025638	Gdpd1	Glycerophosphodiester phosphodiesterase domain containing 1	0.3	0.4	0.5	0.4
NM_026520	2900009I07Rik	RIKEN cDNA 2900009I07 gene	0.8	0.7	0.5	0.7

**Table 15:** List of genes that exhibited the highest ranking at the intermediate time point of 8 week as revealed by the MNI analysis in the muscle of mice.

Gene accession No.	Gene symbol	Description	Fold change (HFD/ND)			
			2wk	4wk	8wk	12wk
**Muscle**						
***Insulin resistance***						
NM_016961	Mapk9	Mitogen activated protein kinase 9	0.8	0.7	0.6	0.8
***Cancer***						
NM_022032	Perp	PERP, TP53 apoptosis effector	0.6	0.7	0.5	0.7
NM_026840	Pdgfrl	Platelet-derived growth factor receptor-like	0.8	0.7	0.7	0.8
***Olfactory transduction***						
NM_146686	Olfr232	Olfactory receptor 232	0.6	0.6	0.6	0.7
NM_146560	Olfr872	Olfactory receptor 872	1.3	1.3	1.3	1.2
***Others***						
NM_027462	Wars2	Tryptophanyl tRNA synthetase 2 (mitochondrial)	0.7	0.8	0.6	0.7
XM_903020	Osbpl7	Oxysterol binding protein-like 7	1.2	1.4	1.6	1.4
XM_484317	EG432803	Predicted gene, EG432803	0.7	0.8	0.6	0.8
NM_001031772	Lin28	Lin-28 homolog B (C. elegans)	0.7	0.7	0.7	0.7
NM_001001602	Dab2ip	Disabled homolog 2 (Drosophila) interacting protein	1.5	1.9	2.2	1.5
NM_145582	Atpbd3	ATP binding domain 3	0.7	0.6	0.6	0.6
AK014527	Cep192	Centrosomal protein 192	1.3	1.4	1.6	1.3
NM_028127	Frmd6	FERM domain containing 6	0.6	0.7	0.6	0.8
NM_172286	6430548M08Rik	RIKEN cDNA 6430548M08 gene	1.2	1.2	1.2	1.1
NM_011105	Pkdrej	Polycystic kidney disease (polycystin) and REJ (sperm receptor for egg jelly, sea urchin homolog)-like	1.5	1.9	2.0	2.2
XM_983096	LOC666331	Hypothetical protein LOC666331	1.2	1.3	1.6	1.4
NM_024196	Tbc1d2	TBC1 domain family, member 20	0.8	0.8	0.7	0.8
NM_198306	Galnt9	UDP-N-acetyl-alpha-D-galactosamine: polypeptide N-acetylgalactosaminyltransferase 9	0.8	0.6	1.0	0.7
AK014852	Glb1	Galactosidase, beta 1 like 3	1.4	1.4	1.4	1.5
NM_001004182	EG434008	Predicted gene, EG434008	0.8	0.7	0.7	0.7
NM_011284	Rpa2	Replication protein A2	1.6	1.5	1.9	1.4
NM_025381	Atp6v1f	ATPase, H+ transporting, lysosomal V1 subunit F	1.4	1.3	1.3	1.4
NM_010571	Irs3	Insulin receptor substrate 3	0.6	0.7	0.6	0.8

**Table 16:** List of genes that exhibited increasing ranking across time points as revealed by the MNI analysis, with a peak at 12 week in the epididymal adipose tissue of mice.

Gene accession No.	Gene symbol	Description	Fold change (HFD/ND)			
			2wk	4wk	8wk	12wk
**Epididymal adipose tissue**						
NM_175236	Adhfe1	Alcohol dehydrogenase, iron containing, 1	0.6	0.6	0.7	0.6
AF015260	Smad7	MAD homolog 7 (Drosophila)	0.7	0.6	0.7	0.5
***Inflammation***						
NM_008035	Folr2	Folate receptor 2 (fetal)	0.7	0.7	0.7	0.6
NM_008802	Pde7a	Phosphodiesterase 7A	2.1	1.9	2.2	2.7
NM_009511	Vipr2	Vasoactive intestinal peptide receptor 2	2.2	2.0	2.0	1.8
***Cancer***						
NM_008586	Mep1b	Meprin 1 beta	0.5	0.5	0.5	0.3
NM_181321	Tmc6	Transmembrane channel-like gene family 6	1.8	1.8	1.7	2.3
***Olfactory transduction***						
NM_008763	Olfr16	Olfactory receptor 16	1.4	2.5	1.2	2.3
NM_001011695	Olfr1000	Olfactory receptor 1000	2.2	2.2	2.5	3.0
***Others***						
NM_139270	Pthr2	Parathyroid hormone receptor 2	2.2	3.1	2.8	2.4
NM_172434	Tnrc4	Trinucleotide repeat containing 4	1.3	1.5	1.4	1.5
NM_173402	Rgs12	Regulator of G-protein signaling 12	0.7	0.7	0.7	0.5
XM_977897	B230217C12Rik	RIKEN cDNA B230217C12 gene	1.8	1.4	1.8	1.8
EG433070	EG433070	Predicted gene, EG433070	1.7	1.8	2.0	2.5
NM_175259	Tmem58	Transmembrane protein 58	1.5	1.3	1.5	1.6
NM_026412	D2Ertd750e	DNA segment, Chr 2, ERATO Doi 750, expressed	0.7	0.6	0.7	0.6
NM_181323	C130090K23Rik	RIKEN cDNA C130090K23 gene	1.5	1.4	1.8	1.5
NM_175406	Atp6v0d2	ATPase, H+ transporting, lysosomal V0 subunit D2	1.4	1.4	1.5	1.3
NM_007725	Cnn2	Calponin 2	0.6	0.6	0.6	0.5
NM_001033274	Brd1	Bromodomain containing 1	1.7	2.2	2.2	2.3

**Table 17:** List of genes that exhibited increasing ranking across time points as revealed by the MNI analysis, with a peak at 12 week in the subcutaneous adipose tissue of mice.

Gene accession No.	Gene symbol	Description	Fold change (HFD/ND)			
			2wk	4wk	8wk	12wk
**Subcutaneous adipose tissue**						
***Inflammation***						
NM_001024852	Auts2	Autism susceptibility candidate 2	0.8	0.9	0.8	0.5
NM_001033536	Rfxdc2	Regulatory factor X domain containing 2 homolog (human)	1.2	1.4	1.3	1.4
NM_009701	Aqp5	Aquaporin 5	0.7	0.7	0.8	0.6
XM_285901	Gm792	Gene model 792, (NCBI)	0.7	0.6	0.5	0.4
NM_019775	Cpb2	Carboxypeptidase B2 (plasma)	1.7	1.6	1.5	2.5
***Cancer***						
NM_009050	Ret	Ret proto-oncogene	1.2	1.3	1.2	1.5
***Olfactory transduction***						
NM_146604	Olfr716	Olfactory receptor 716	1.9	1.7	2.0	3.1
NM_146963	Olfr45	Olfactory receptor 45	1.6	1.5	1.9	2.2
***Others***						
NM_178046	Svil	Supervillin	0.7	0.8	0.7	0.6
NM_018764	Pcdh7	Protocadherin 7	1.5	1.4	1.3	1.8
NM_030061	Spink12	Serine peptidase inhibitor, Kazal type 12	1.4	1.4	1.3	1.7
NM_178741	Klhl8	Kelch-like 8 (Drosophila)	1.3	1.3	1.3	1.6
NM_175696	C530028O21Rik	RIKEN cDNA C530028O21 gene	1.3	1.3	1.2	1.5
NM_177711	4932411G14Rik	RIKEN cDNA 4932411G14 gene	0.7	0.6	0.7	0.5
NM_181579	Pof1b	Premature ovarian failure 1B	1.7	1.8	1.6	2.2
XM_489612	4921511H13Rik	RIKEN cDNA 4921511H13 gene	1.3	1.4	1.2	1.6
NM_172456	Endogl1	Endonuclease G-like 1	1.6	1.9	1.4	2.2
NM_001033488	Gm1964	Gene model 1964, (NCBI)	1.5	1.4	1.5	1.5
NM_181318	Rasgef1b	RasGEF domain family, member 1B	0.7	0.8	0.8	0.6
NM_183017	Ttll12	Tubulin tyrosine ligase-like family, member 12	0.8	0.6	0.7	0.7
NM_010075	Dpp6	Dipeptidylpeptidase 6	1.1	1.2	1.2	1.3
NM_026175	Sf3a1	Splicing factor 3a, subunit 1	1.5	1.4	1.7	1.9

**Table 18:** List of genes that exhibited increasing ranking across time points as revealed by the MNI analysis, with a peak at 12 week in the muscle of mice.

Gene accession No.	Gene symbol	Description	Fold change (HFD/ND)			
			2wk	4wk	8wk	12wk
**Muscle**						
***Insulin resistance***						
NM_145435	Pyy	Peptide YY	1.4	1.4	1.3	1.5
***Inflammation***						
NM_138648	Olr1	Oxidized low density lipoprotein (lectin-like) receptor 1	0.5	0.6	0.5	0.3
NM_009132	Scin	Scinderin	0.6	0.6	0.7	0.7
***Cancer***						
NM_145833	Lin28	Lin-28 homolog (C. elegans)	0.7	0.7	0.7	0.7
NM_010358	Gstm1	Glutathione S-transferase, mu 1	1.3	1.4	1.4	1.3
NM_001029979	Safb2	Scaffold attachment factor B2	0.7	0.7	0.7	0.6
***Olfactory transduction***						
NM_146750	Olfr689	Olfactory receptor 689	0.6	0.7	0.7	0.5
NM_146943	Olfr347	Olfactory receptor 347	1.2	1.3	1.2	1.5
***Others***						
NM_009372	Tgif1	TG interacting factor 1	1.2	1.1	0.8	0.6
XM_887155	Igsf10	Immunoglobulin superfamily, member 10	0.7	0.5	0.6	0.4
NM_009418	Tpp2	Tripeptidyl peptidase II	1.2	1.4	1.2	1.5
NM_008118	Gif	Gastric intrinsic factor	0.8	0.7	0.7	0.8
NM_026972	Cd209b	CD209b antigen	0.9	0.8	0.6	0.7
NM_145358	Camkk2	Calcium/calmodulin-dependent protein kinase kinase 2, beta	1.2	1.2	1.3	1.2
BC051526	Cd22	CD226 antigen	0.8	0.9	0.9	0.7
NM_029946	4931407K02Rik	RIKEN cDNA 4931407K02 gene	1.3	1.1	1.2	1.3
NM_172652	4632411B12Rik	RIKEN cDNA 4632411B12 gene	1.7	1.4	1.4	1.9
NM_011598	Fabp9	Fatty acid binding protein 9, testis	0.8	0.8	0.9	0.7
NM_011023	Otx1	Orthodenticle homolog 1 (Drosophila)	0.7	0.7	0.7	0.5
NM_019579	Mpp5	Membrane protein, palmitoylated 5	0.5	0.5	0.6	0.3
NM_007555	Bmp5	Bone morphogenetic protein 5	0.9	0.9	0.8	0.8
NM_001039047	Trim58	Tripartite motif-containing 58	1.3	1.2	1.5	1.5
NM_020049	Slc6a14	Solute carrier family 6 (neurotransmitter transporter), member 14	0.8	0.6	0.7	0.6

**Table 19:** List of genes that exhibited a constant high MNI ranking throughout the time course as revealed by the MNI analysis in the peripheral tissues of mice.

Gene accession No.	Gene symbol	Description	Fold change (HFD/ND)			
			2wk	4wk	8wk	12wk
**Epididymal adipose tissue**						
***Insulin resistance***						
NM_001039138	Camk2g	Calcium/calmodulin-dependent protein kinase II gamma	0.6	0.5	0.6	0.5
***Others***						
NM_145463	Tmem46	Transmembrane protein 46	0.6	0.6	0.7	0.6
**Subcutaneous adipose tissue**						
***olfactory transduction***						
NM_207566	Olfr1173	Olfactory receptor 1173	2.0	2.3	1.9	2.7
NM_146709	Olfr411	Olfactory receptor 411	1.5	1.4	1.4	1.6
NM_146524	Olfr855	Olfactory receptor 855	1.7	1.6	2.0	1.6
***Others***						
NM_178110	Trim62	Tripartite motif-containing 62	1.6	1.8	1.6	1.8
**Muscle**						
***Cancer***						
NM_024124	Hdac9	Histone deacetylase 9	0.6	0.5	0.6	0.6
***Others***						
NM_177861	Tmem67	Transmembrane protein 67	0.7	0.6	0.7	0.5

## Discussion

Animals use their olfactory system to monitor the chemical environment for molecules that reveal food sources or toxic substances and signal the presence of predators [11]. There are numerous olfactory receptors of different types, with as many as 1,000 in the mammalian genome that represent approximately 3% of the human genomeentire genetic information [12]. The information related to odor gathered by these olfactory receptors is funneled through a common signaling pathway. When an olfactory receptor binds to its odorant, it activates a single species of G protein, the olfactory trimeric G protein (Golf), which in turn activates the olfactory isoform of adenylate cyclase (AC3) [12]. Converging evidence has demonstrated that the olfactory system is a target for hormones related to metabolism and food-intake regulation; it adapts its function to nutritional needs by promoting or inhibiting food foraging [13]. Recent studies have found that obese patients display decreased olfactory acuity [14] and are significantly more likely to have absolute olfactory dysfunction or anosmia [15]. Furthermore, Simchen et al. showed that the abilities to detect and identify odors have been found to decrease as body mass index (BMI) increases in subjects less than 65 years old, independent of any linkage to food odor or gender [16]. Recently, the elements of olfactory-like chemosensory signaling have been found to also present in nonolfactory tissues such as testis [17], brain [18], and heart [19]. To our knowledge, this is the first study that shows a differential mRNA expression and high MNI ranking of olfactory receptors in the epididymal and subcutaneous fat tissues and muscles between ND and HFD-fed mice (Tables 1, 2, 3). These results imply that the olfactory receptors and the molecules involved in olfactory transduction might be the mediators of HFD-induced obesity progression in the peripheral tissues. This hypothesis is supported by the fact that the increased cAMP production by AC3 activates cAMP responsive element binding (CREB) protein, leading to increased adipogenesis in an obese mouse model. Furthermore, mice lacking AC3, which is a downstream regulator of olfactory receptors, exhibit obesity that is apparently caused by low locomotor activity, hyperphagia, and leptin insensitivity [20]. In future studies, it will be intriguing to further investigate the role of individual olfactory receptors in peripheral tissues, such as the pancreas, liver, muscle, and fat, to better understand the activation process of these signaling pathways and their physiological roles.

Cancer-related genes such as *Nek11*, *A4gnt*, *Srp9*, *Gli2*, *Gucy2c*, *Lsm1*, *Duoxa1*, *Lasp1*, *Ret*, *Bex2*, *Vav3*, *Kcnrg*, *Tle6*, *Rab23*, *Dcc*, *Rassf2*, *Perp*, *Pdgfr1*, *Lin28*, *Gstm1*, *Safb2*, *Tmem46*, and *Hdac9* were remarkably overrepresented in time-course clusters identified by the MNI analysis in the epididymal and subcutaneous fat tissues and gastrocnemius muscle of mice with diet-induced obesity (Figs. 4, 5, 6, 7, 8). Of these 23 genes, 6 are known breast cancer-related genes (*Lsm1*, *Duoxa1*, *Ret*, *Bex2*, *Rassf2*, and *Safb2*). *Lsm1* is a transforming oncogene that is amplified and overexpressed in breast cancer [21] and might affect either cell cycle progression or apoptosis [22]. *Duoxa1*, which was originally identified as a numb-interacting protein, was recently shown to function as a maturation factor in breast cancer [23]. *Ret* exhibits both estrogen- and retinoic acid-dependent transcriptional modulation in breast cancer [24]. *Bex2* has a significant role in promoting cell survival and growth in breast cancer cells [25], [26], and *Rassf2* might function as a tumor suppressor gene in *in vitro* cell migration and cell cycle progression [27]. The expression of Safb2 protein, which functions as estrogen receptor co-repressor and growth inhibitor, was lost in approximately 20% of breast cancers [28]. Many studies have attempted to determine the relationship between diet and breast cancer. Dietary fat is a source of endogenous estrogen and has been suggested as a possible risk factor for breast cancer [29]. To our knowledge, this is the first study showing an association between these 6 genes involved in breast cancer development and HFD-induced obesity in a rodent model.

Colon cancer-related genes such as *Nek11*, *Gucy2c*, *Srp9*, *Tle6*, and *Pdgfrl* were also overrepresented in the time-course clusters identified by the MNI analysis in the epididymal and subcutaneous fat tissues and gastrocnemius muscle of mice with diet-induced obesity (Figs. 4, 5, 6, 7, 8). *Nek11*, a member of the NIMA-related kinase family, phosphorylates Cdc25a and controls its degradation; Cdc25a phosphorylation is required for cell cycle progression in colorectal cancer cells [30]. *Gucy2c* and *Srp9* have been shown to be overexpressed in colorectal cancer cells and were recently shown to function as a candidate biomarker for colon cancer [31], [32]. *Tle6* is recurrently overexpressed in human colon cancer and enhances cell proliferation, colony formation, migration, and xenograft tumorigenicity [33]. *Pdgfrl* acts as a tumor suppressor and inhibits the growth of colorectal cancer cells [34]. Epidemiological studies indicate that both high body weight and high body mass index (BMI) were significantly associated with an increased colon cancer risk. Intra-abdominal visceral obesity, high plasma glucose levels, HbA1C, and C-peptide were also found to be associated with increased risk of colorectal cancer [35]–[38]. The current study showed that the above-mentioned genes that are involved in the regulation of colon cancer might play a genetic role in the development of obesity. No mechanistic insights have been reported to explain the relationship between the regulation of cancer-related genes in the adipose tissue or muscle and cancer susceptibility. It could be probable that the changes in the expression of cancer-related genes in the adipose tissue may accompany the regulation of same genes in epithelial tissues such as breast or colon.

Genes that were found to have the highest rank at the early phase and return to baseline after several weeks might be considered genetic mediators of acute-phase response in metabolic processes related to HFD-induced obesity. *Dusp12* was one of the 58 genes that were observed to have decreasing ranking during the development of obesity, with a peak at 2 week. Previous studies identified several single nucleotide polymorphisms in this gene associated with type 2 diabetes in different populations, including Caucasians and Chinese [39]. *Dusp12* is a glucokinase-associated protein that participates in glycolysis in the liver and dephosphorylation of cytoplasmic glucokinase in the pancreatic beta cells [40]. Therefore, *Dusp12* might play a role in the regulation of glycolysis during the early stages of obesity. When glycolysis was decreased, whole-body glucose disposal was also reduced, indicating a decrease in glucose utilization in the peripheral tissues in response to the HFD. The latter likely results from an impaired glucose transport that precedes impaired insulin signaling.

*Def6* and *Mapk9* were one of the 145 genes that were found to have the highest rank at the intermediate time points of 4 or 8 weeks during the development of obesity. *Def6*, a novel type of activator for Rho GTPase, is expressed in myeloid cells, and disruption of Def6 expression leads to defects in toll-like receptor 4 (TLR4) signaling and innate immune responses [41]. Rho GTPases have been shown to be recruited to the cytosolic domain of TLR and the closely related interleukin 1 receptor (IL-1R) and to regulate the production of proinflammatory cytokines [42], [43]. In the present study, the high MNI ranking of *Def6* in the subcutaneous adipose tissue of HFD-fed mice suggested that it might participate in the regulation of obesity-induced inflammation through TLR4 signaling. *Mapk9*, which is ubiquitously expressed, can invoke transcription factors such as c-Jun and many other apoptosis-related proteins [44]. Interestingly, recent studies have shown that the knockdown of *Mapk9* leads to reduced serum levels of glucose, insulin, and homeostatic model assessment and therefore reverses insulin resistance in HFD-fed mice [45]. These findings provide supporting evidences to the high MNI ranking of *Mapk9* associated with HFD-induced obesity observed in the present study. However, further studies are required to elucidate the precise function of *Mapk9* in the development of HFD-induced type 2 diabetes.

*Smad7*, *Adhfe1*, and *Pyy* are one of the 65 genes that showed increasing ranking during the development of obesity, with a peak at 12 weeks. *Smad7* was initially characterized as a factor induced by shear stress in vascular endothelial cells [46]. Only recently, new functions of *Smad7* were elucidated: it inhibits transforming growth factor-β (TGF-β)-activated responses [46]. TGF-β is known to inhibit adipose differentiation of preadipocyte cell lines and primary cultures [47] and to block adipogenesis *in vivo* [48]. This suggests that *Smad7* enhances adipogenesis through the inhibition of TGF-β signaling. *Adhfe1* was characterized as a hydroxyacid-oxoacid transhydrogenase that catalyzes the conversion of γ-hydroxybutyrate to succinic semialdehyde [49]. Recently, *Adhfe1* was suggested to play a role in adipocyte differentiation. The expression of Adhfe1 transcript is tightly linked to the phenotype of mature adipocytes both *in vivo* and *in vitro*, although the mechanisms underlying *Adhfe1*-mediated regulation of adipogenesis remain poorly understood [50]. *Pyy*, which is expressed and secreted in endocrine intestinal cells, plays a role in reducing appetite and caloric intake [51]. Recently, plasma Pyy concentrations were found to be decreased in both obese humans [52] and diet-induced obese mice [53]. These studies might suggest that *Smad7* and *Adhfe1* play a role in obesity by amplifying the aggressive effect of adipogenesis.

*Camk2g* and *Tmem67* are one of the 8 genes that exhibited a constant high MNI ranking from 2 to 12 weeks. The increase of cytosolic Ca^2+^ in the beta cells is central to the initiation of insulin secretion under physiological conditions [54]. Recent findings suggest that *Camk2g* involved in the regulation of calcium in the islet beta cells is a candidate gene for type 2 diabetes [55]. The *Tmem67* gene mediates a fundamental developmental stage of ciliary formation and epithelial morphogenesis [56]. In addition, defects in the *Tmem67* gene resulted in Meckel syndrome type 3, Joubert syndrome type 6, and nephronophthisis 11, which show many clinical phenotypic similarities, including hepatic fibrosis [56], [57]. Consumption of fat-rich diets seems to play an important role in the pathogenesis of hepatic steatosis and its progression to fibrosis [58]. The constantly high MNI ranking of *Tmem67* from 2 to 12 weeks associated with a HFD suggests that *Tmem67* might participate in the development of hepatic fibrosis.

In summary, this study is the most comprehensive investigation of the gene expression patterns conducted using a time-resolved approach to gain insight into the development of HFD-induced obesity in a mouse model. A reverse-engineered gene network was used for the first time for the identification of key genetic mediators and pathways that have been implicated in the initiation and advancement of obesity. We highlighted the sequential induction of distinct olfactory receptors and stimulation of cancer-related genes during the development of obesity. To our knowledge, the proposed changes in the olfactory transduction machinery as per the MNI ranking have not been previously reported. These putative mechanisms clearly need further investigation. The top 5 genes recognized through the MNI analysis at each time points (2, 4, 8, and 12 weeks) and gene clusters identified based on their temporal patterns in the 3 different tissues (visceral and subcutaneous adipose tissues and muscle) of mice need special attention as potential genetic mediators for obesity progression.

## Supporting Information

Figure S1 **The basal expression levels of some target genes identified by MNI analysis.** Quantitative real-time PCR analysis of the basal expression on highly ranked olfactory genes and top 5 genes at week 4 in the epididymal adipose tissues of (A) ND- or (B) HFD-fed mice. Results are presented as the average ± SEM of at least 3 separate experiments.
(TIF)
