# Supplementary figures and images for: Induction of Olfaction and Cancer-Related Genes in Mice Fed a High-Fat Diet as Assessed through the Mode-of-Action by Network Identification Analysis
Source: PLoS One. 2013 Mar 26;8(3):e56610. doi: 10.1371/journal.pone.0056610 (PMC3608641; doi:10.1371/journal.pone.0056610)

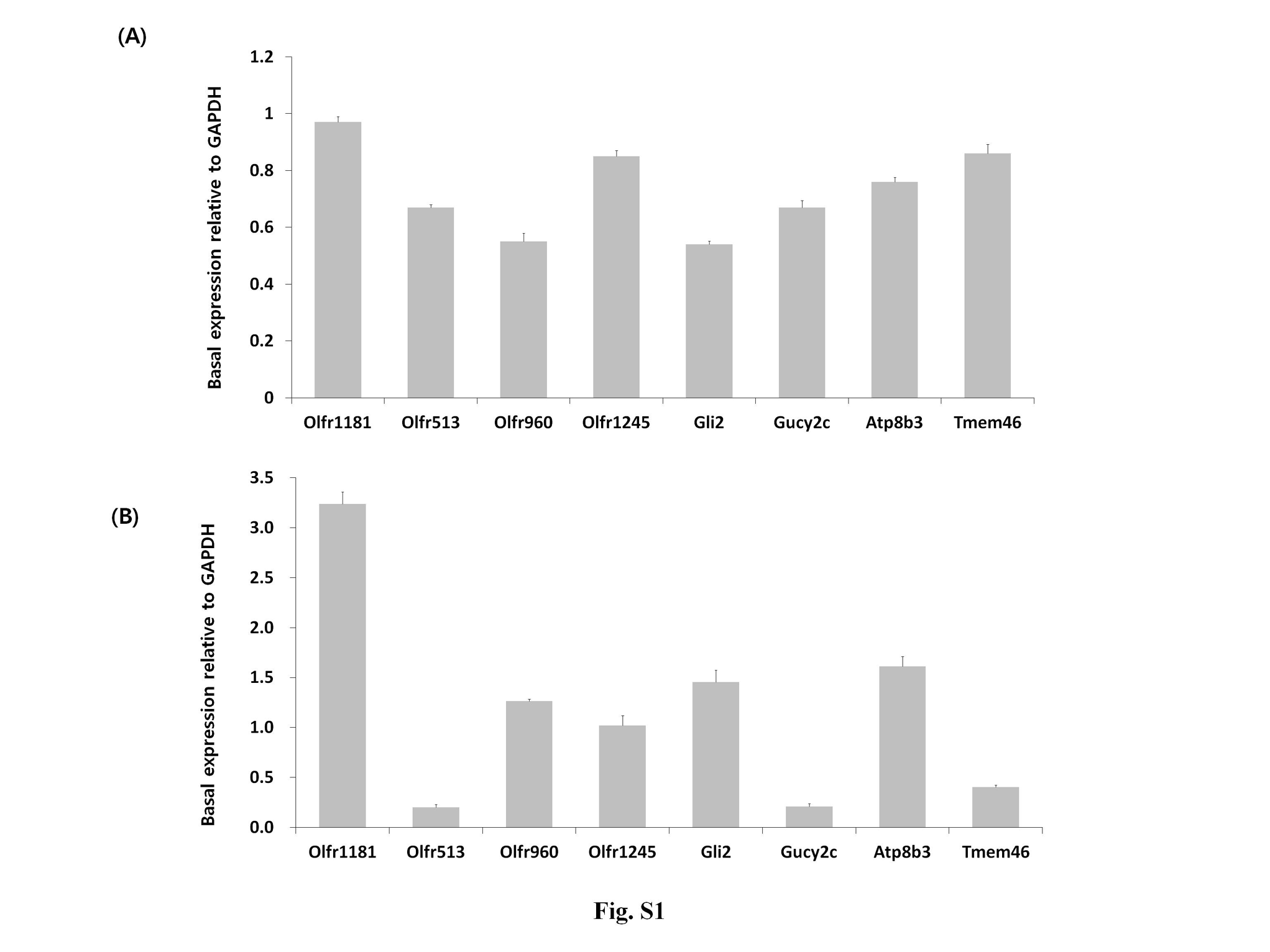

Supplement: Figure S1 — The basal expression levels of some target genes identified by MNI analysis. Quantitative real-time PCR analysis of the basal expression on highly ranked olfactory genes and top 5 genes at week 4 in the epididymal adipose tissues of (A) ND- or (B) HFD-fed mice. Results are presented as the average ± SEM of at least 3 separate experiments. (TIF) [file pone.0056610.s001.tif]
